# Supplementary material for: Radical Dehalogenation and Purine Nucleoside Phosphorylase E. coli: How Does an Admixture of 2′,3′-Anhydroinosine Hinder 2-fluoro-cordycepin Synthesis
Source: Biomolecules. 2021 Apr 7;11(4):539. doi: 10.3390/biom11040539 (PMC8067715; doi:10.3390/biom11040539)
Supplement: Supplementary file 1 [file biomolecules-11-00539-s001.pdf]

Supplementary Materials

Radical Dehalogenation and Purine Nucleoside Phosphorylase *E. coli*: How does an Admixture of 2',3'-Anhydroinosine Hinder 2-fluoro-cordycepin Synthesis

Alexey L. Kayushin [1], Julia A. Tokunova [1], Ilja V. Fateev [1], Alexandra O. Arnautova [1], Maria Ya. Berzina [1], Alexander S. Paramonov [1], Olga I. Lutonina [1], Elena V. Dorofeeva [1], Konstantin V. Antonov [1], Roman S. Esipov [1], Igor A. Mikhailopulo [2], Anatoly I. Miroshnikov [1] and Irina D. Konstantinova [1,*]

Content	Page number
**Figure S1.** The HPLC profile of the reaction mixture of 2-fluorocordycepin synthesis	S-2
**Figure S2.** A mass-spectrum of unknown nucleoside (**10**)	S-2
**Figure S3.** The data of LC-MS 3′-dIno (**9**) with 16% of compound (**11**)	S-3
**Figure S4.** The HPLC profile of the reaction mixture obtained during 2-fluorocordycepin synthesis. Synthesis of 2-F-Cord.	S-4
**Figure S5.** HPLC profile of the reaction mixture obtained during hydrolysis of epoxide **11** in 5 mM potassium-phosphate buffer (pH 7.0)	S-5
**Figure S6.** HPLC profile of the reaction mixture obtained during hydrolysis of **11** in 5 mM potassium-phosphate buffer (appeared pH 7.0), D_2O	S-5
**Figure S7.** HPLC profile of the reaction mixture obtained during hydrolysis of **11** in 5 mM potassium-phosphate buffer (appeared pH 4.1), D_2O	S-5
**Figure S8.** A fragment of ^{1}H NMR spectrum of **12**<br>**Figure S9.** A fragment of ^{2}H NMR spectrum of nucleoside **12**	S-6
**Figure S10.** A fragment of ^{13}C NMR spectrum of compound **12**	S-7
**NMR data** for nucleosides **10, 11, 12, 14**	S-8 – S-31
The ^{1}H NMR spectrum of compound **13** (DMSO-d6, 30 °C).	S-32

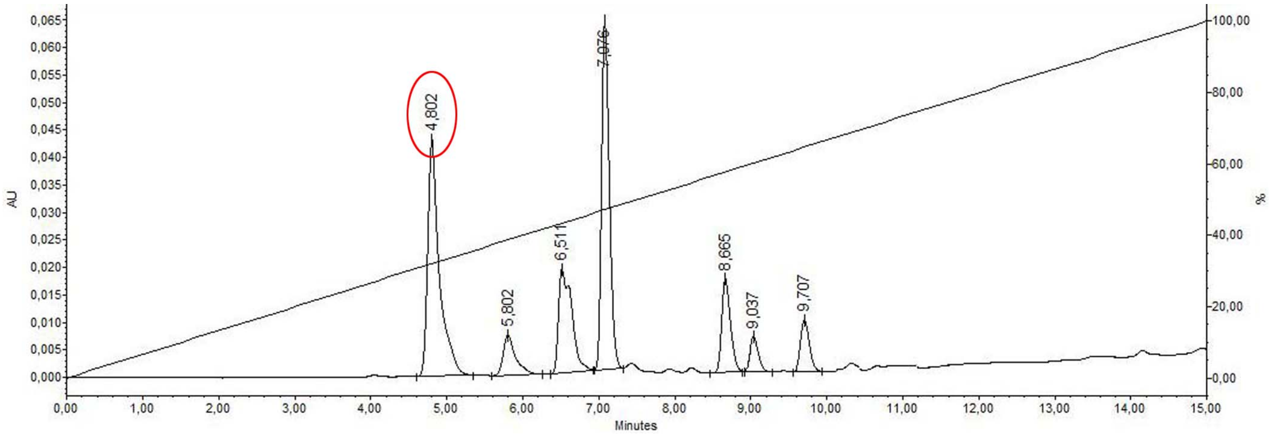

Comp	RT (min)	% Area
?	4.802	31.39
Hyp	5.802	5.45
Ino	6.511	15.50
3'-dIno	7.076	30.40
2-F-Ade	8.655	8.93
2-F-Ado	9.037	3.23
2-F-Cord	9.707	5.10

Figure S1. The HPLC profile of the reaction mixture of anomal 2-fluorocordycepin synthesis (14 days after start of the reaction). Reaction conditions: 1.5 mM 3'-dIno, 1 mM 2-F-Ado, 2 mM potassium phosphate (pH 7.0), 100 mL, 50 ° C, 580 units of PNP.

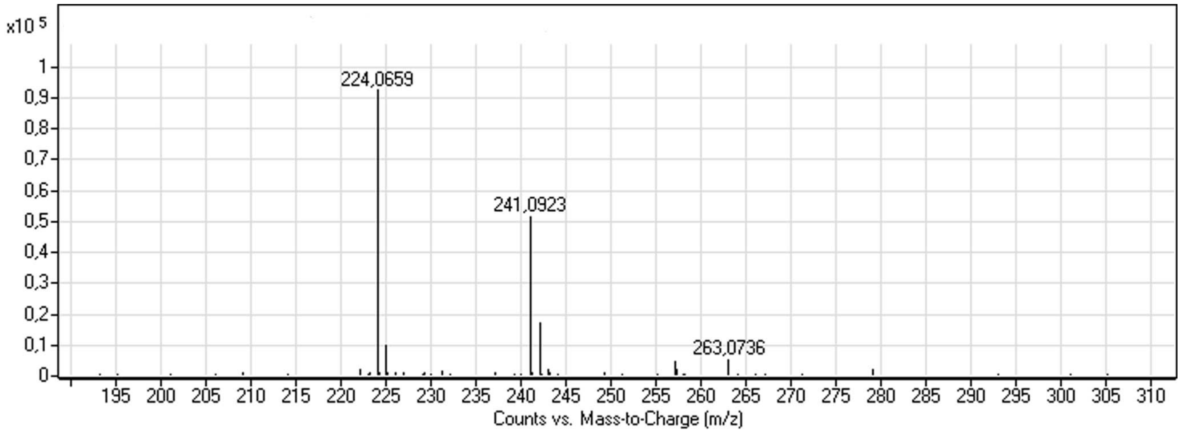

$[M + H]^{+,}$ found (calculated)	Other, found (calculated)
241.0923 (241.0937, $C_9H_{13}N_4O_4$)	$[M + Na]^+$ 263.0736 (263.0756, $C_9H_{12}N_4NaO_4$)

Figure S2. A mass-spectrum of unknown nucleoside (**10**) (Figure SI-1, RT = 4.802 min).

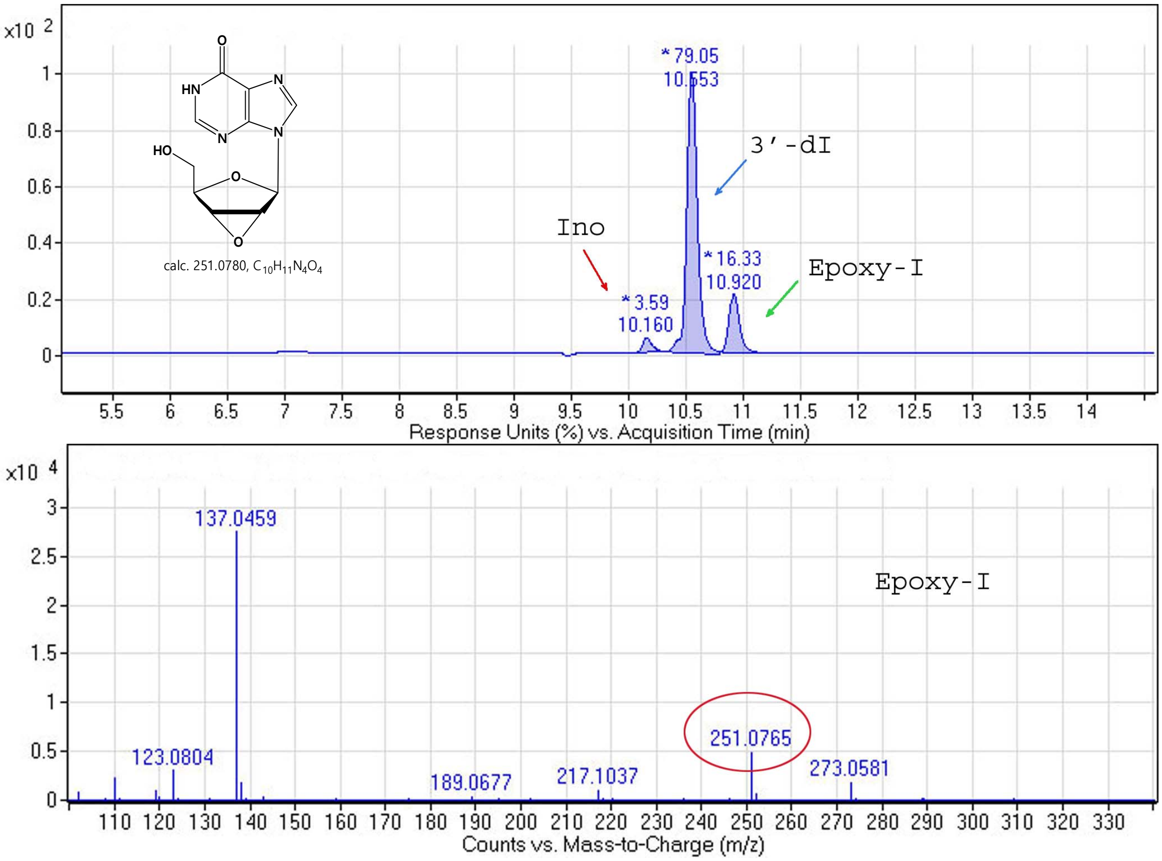

Figure S3. The data of LC-MS 3′-dIno (**9**) with 16% of compound (**11**) obtained as shown on Scheme **1**, route **A**.

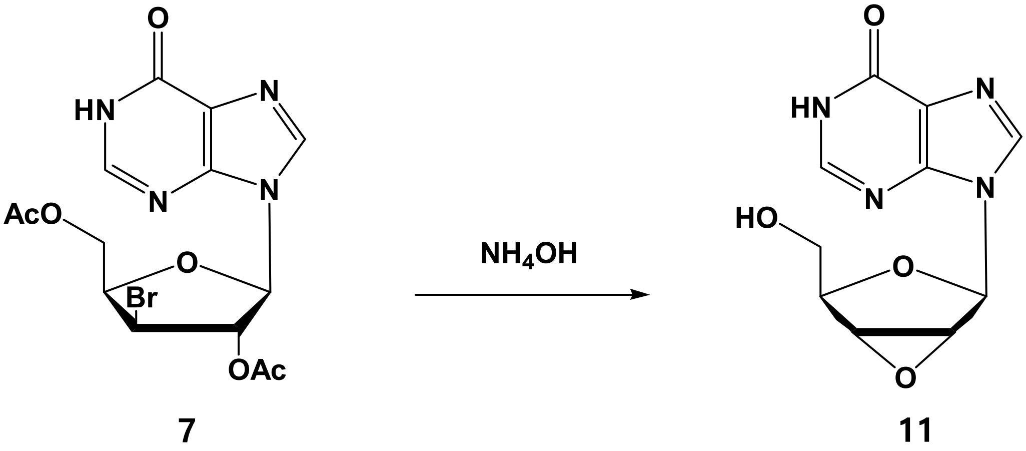

Scheme 1. Synthesis of 2',3'-anhydroinosine (**11**).

Synthesis of *2-F-Cord.*

3'-dIno was obtained using ***catalytic dehalogenation*** (Scheme 1, route **B**).

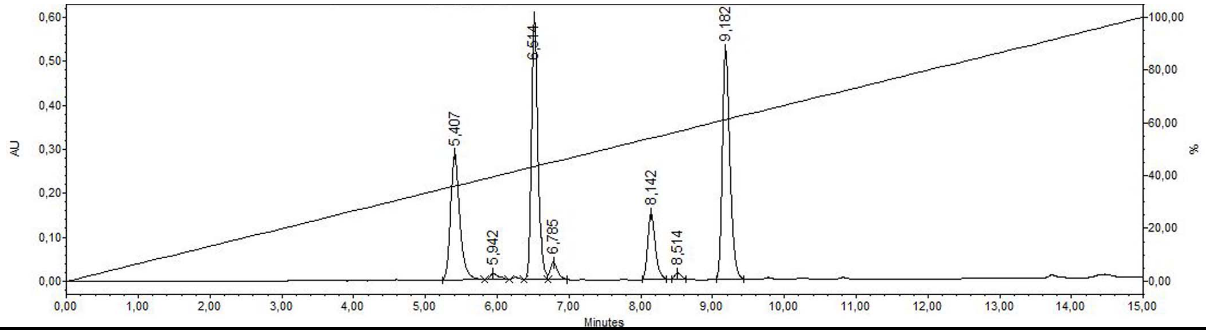

Comp.	RT (min)	% Area
Hyp	5.407	22.39
Ino	5.942	1.08
3'-dIno	6.514	32.58
2-F-Ade	8.142	9.08
2-F-Ado	8.514	0.68
2-F-Cord	9.182	31.93

Figure S4. The HPLC profile of the reaction mixture obtained during 2-fluorocordycepin synthesis (16 days after start of the reaction). 3'-dIno was obtained using ***catalytic dehalogenation (Scheme 1, route B)***. Reaction conditions: 1.5 mM 3'-dIno, 1 mM 2-F-Ado, 2 mM potassium phosphate (pH 7.0), 1.0 mL, 50 °C, 12.5 units of PNP.

9-(3-Deoxy-β-D-ribofuranosyl)-2-fluoroadenine (2-F-Cord)

2-Fluoroadenosine [1] (0.29 g, 1 mmol) was dissolved in 1 L of water under heating and stirring. The solution was cooled to 50 °C, 0.38 g (1.5 mmol) 3'-deoxyinosine **9** and 0.27 g (2 mmol) of KH_2PO_4 were added, and the pH of the mixture was adjusted to 7.0; PNP (12500 IU) was added and the reaction mixture was incubated at 52 °C for 20 days under stirring. The desired product was isolated as described above [2]. Yield 193 mg (0.72 mmol, 72%) as lyophilized powder; purity 99.78 % (R_t = 9.18 min, method II). UV (H_2O, pH 7.0) λ_{max}, nm (ε, $M^{-1}cm^{-1}$): 261 (14800); λ_{min}, nm (ε, $M^{-1}cm^{-1}$): 225 (5100). ESI-MS data, ^1H and ^{13}C NMR spectra were described in [2].

1 V.B. Berzin, et all. The preparative method for 2-fluoroadenosine synthesis. *Russ. J. Bioorg. Chem.* **2009**, *35* (2), 193–196; doi:10.1134/S1068162009020071

2 A.O. Denisova, et all. The chemoenzymatic synthesis of 2-chloro- and 2-fluorocordycepins. *Synthesis*, **2017**, *49*, 4853–4860.

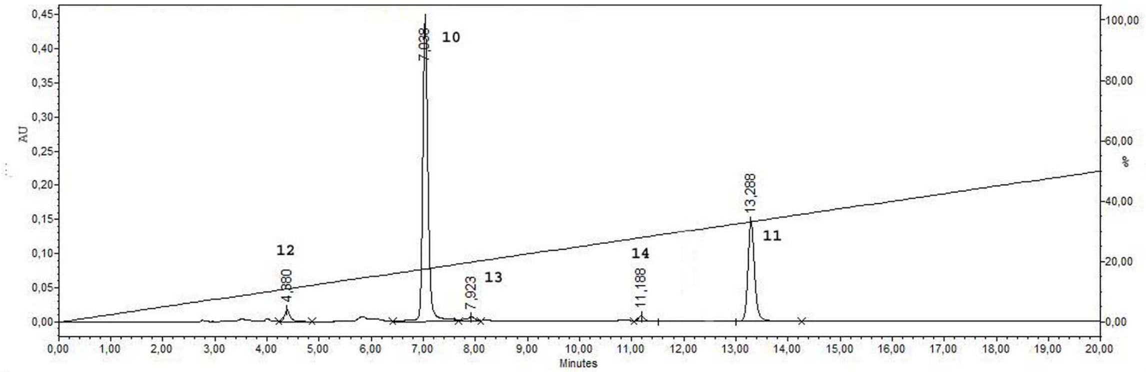

Figure S5. HPLC profile of the reaction mixture obtained during hydrolysis of epoxide **11** in 5 mM potassium-phosphate buffer (pH 7.0), 96 h after reaction start, detection at 260 nm.

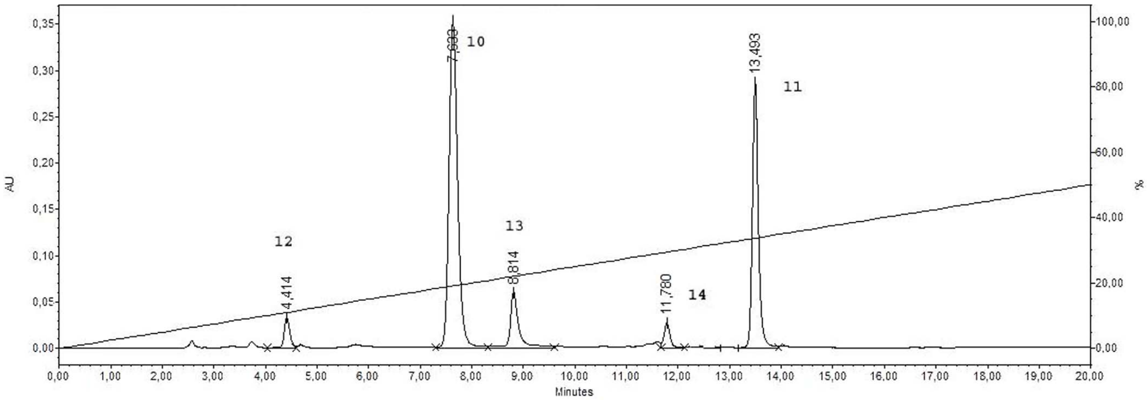

Figure S6. HPLC profile of the reaction mixture obtained during hydrolysis of **11** in 5 mM potassium-phosphate buffer (appeared pH 7.0), D_2O, 96 h after reaction start, detection at 260 nm.

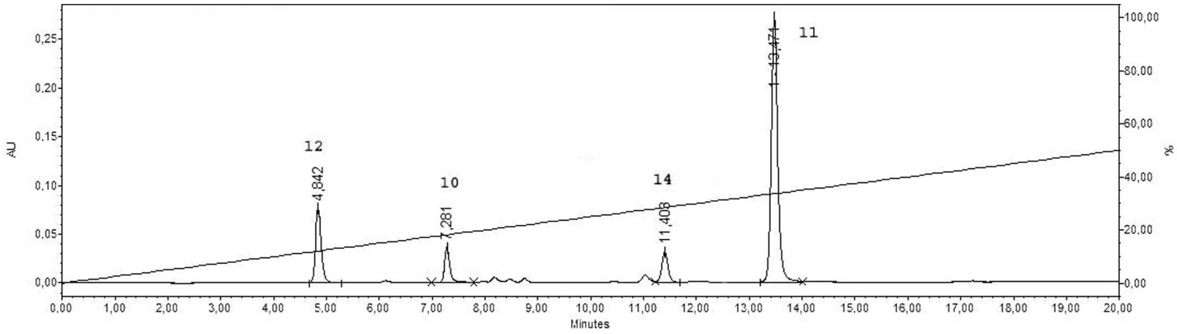

Figure S7. HPLC profile of the reaction mixture obtained during hydrolysis of **11** in 5 mM potassium-phosphate buffer (appeared pH 4.1), D_2O, 96 h after reaction start, detection at 260 nm.

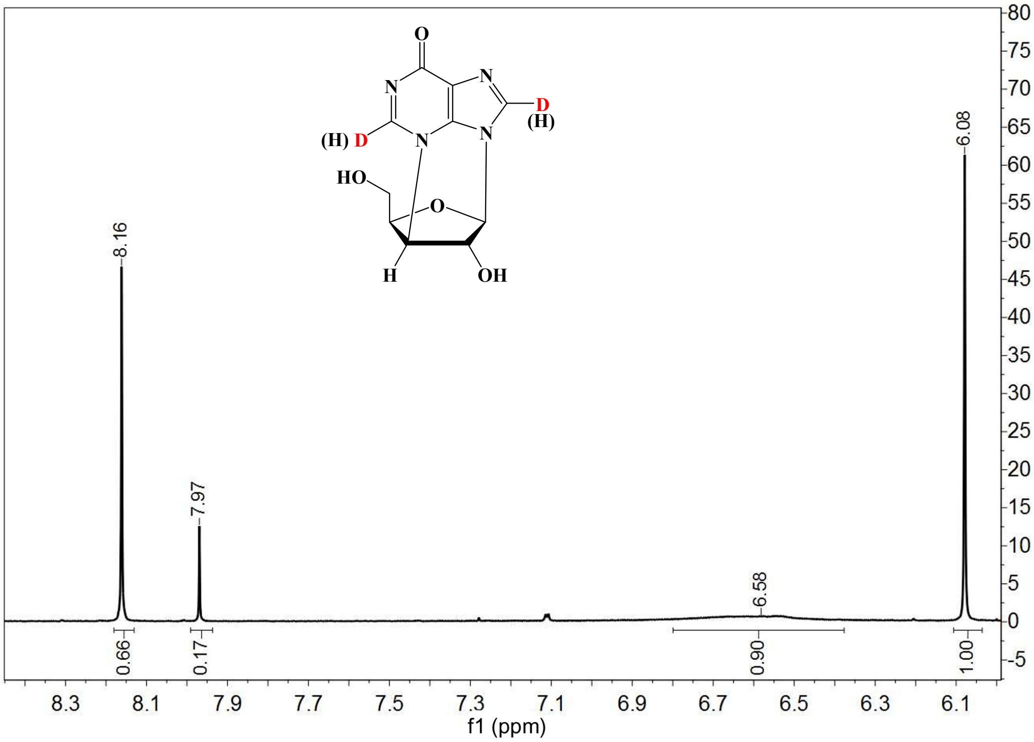

Figure S8. A fragment of ^{1}H NMR spectrum of **12**.

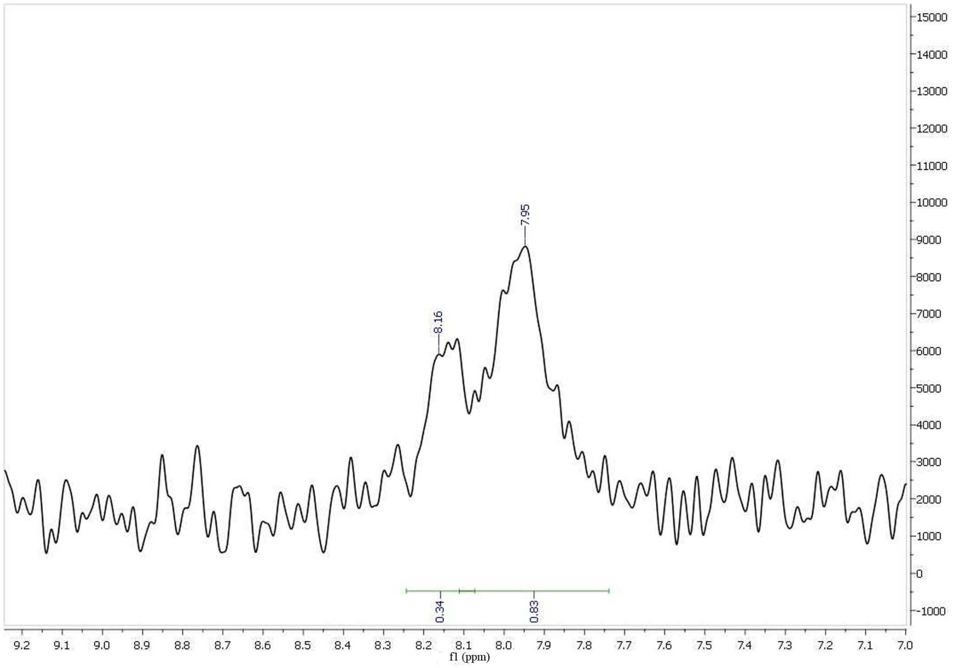

Figure S9. A fragment of ^{2}H NMR spectrum of nucleoside **12**.

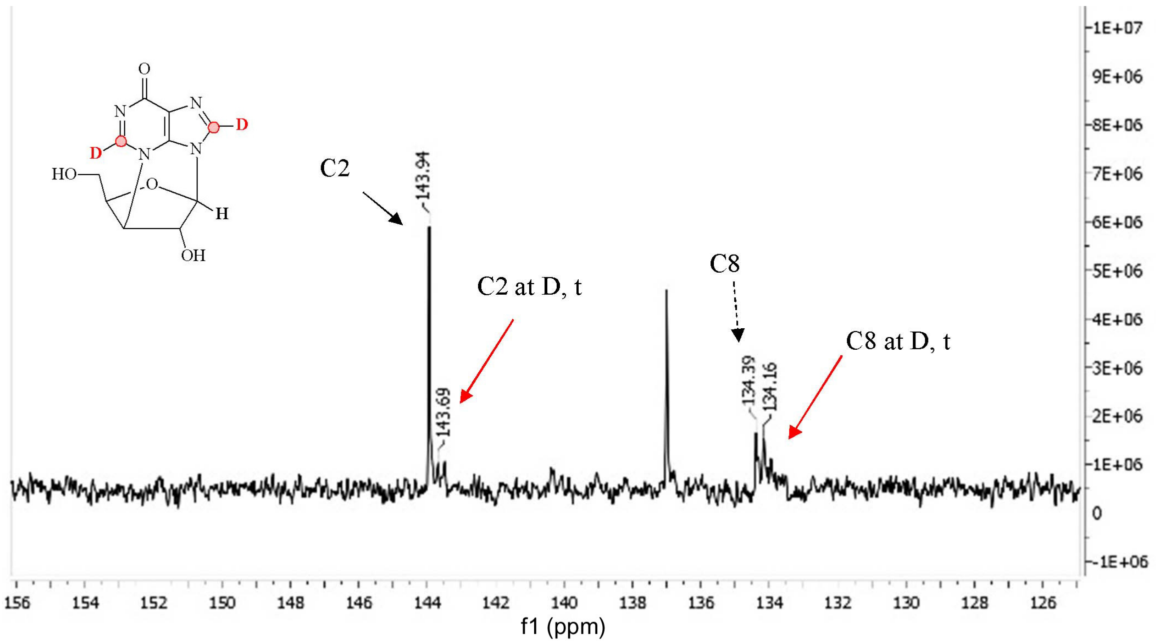

Figure S10. A fragment of ^{13}C NMR spectrum of compound **12**.

NMR data

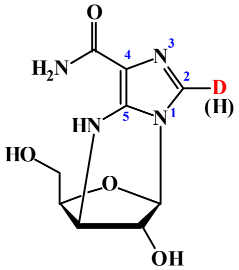

Nucleoside 10: HPLC: R*t*= 7.38 min, purity 96.6%. UV/Vis (D_2O, appeared pH 4.1): λ_{max}, nm (ε, $M^{-1}cm^{-1}$): 276 (14900), λ_{min}, nm (ε, $M^{-1}cm^{-1}$): =222 (2900). HRMS (ESI^-): (*m*/*z*) [$(M + H)^+$] - calculated for $C_9H_{13}N_4O_4$ 241.0931, found 241.0955; calculated for $C_9H_{12}DN_4O_4$ 242.0994, found 242.1026.

^{1}H NMR (700 MHz, DMSO-d6, 30 °C): sδ = 7.12 (s, 0.18 H, H-8), 6.69 and 6.61 (2 s, 2 H, $CONH_2$), 6.39 (d, *J* = 4.4 Hz, 1 H, NH), 5.57 (d, *J* < 0.5 Hz, 1H, H-1′), 4.71 (br. sign, 0.36 H, OH-5′), 4.48 (br. sign, 1H, H-2′), 4.25 (dt, *J* = 3.2, 6.6, 6.6 Hz, 1 H, H-4′), 3.70 (m, 1 H, H-3′), 3.54 (dd, *J* = 7.0, 11.3 Hz, 1 H, CH-5′a), 3.46 ppm (dd, *J* = 6.5, 11.3 Hz, 1 H, CH-5′b).

^{2}H NMR (700 MHz, DMSO-d6, 30 °C): δ = 7.12 ppm (s, D-8).

^{13}C NMR (176 MHz, DMSO-d6, 30 °C): δ = 165.46 (C=O), 139.19 (C5), 127.03(C2), 126.79 (t, *J* = 18.5Hz, C2-D), 110.91 (C4), 85.14 (C4′), 83.96 (C1′), 73.09 (C2′), 60.07 (C5′), 56.16 ppm (C3′).

^{15}N NMR (71 MHz, DMSO-d6, 30 °C): δ = 253.35 (N3), 177.94 (N1), 95.31 (NH_2), 56.88 ppm (NH).

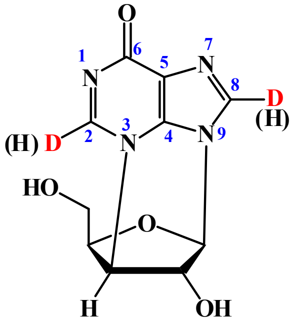

Nucleoside 12: HPLC: R*t* = 5.01 min, purity 100%. UV/Vis (D_2O, appeared pH 4.1): λ_{max}, nm (ε, $M^{-1}cm^{-1}$): 254 (9900), λ_{min}, nm (ε, $M^{-1}cm^{-1}$): =229 (3500). HRMS (ESI^-): (*m*/*z*) [$(M + H)^+$] - calculated for $C_{10}H_{11}N_4O_4$ 251.0775, found 251.0797; calculated for $C_{10}H_{10}DN_4O_4$ 252.0838, found 252.0868; calculated for $C_{10}H_9D_2N_4O_4$ 253.0900, found 253.0914.

^{1}H NMR (700 MHz, DMSO-d6, 30 °C): δ = 8.16 (s, 0.66 H, H-8), 7.97 (s, 0.18 H, H-2), 6.08 (1H, d, *J J* < 1 Hz, H-1′), 5.07 (d, *J* = 3.1 Hz, 1 H, H-3′), 4.85 (m,1 H, *J* < 1 Hz, H-2′), 4.62 (ddd, *J* = 3.4, 6.1, 7.5 Hz.0, 1 H, H-4′), 3.41 (dd, *J* = 6.1, 11.1 Hz, 1 H, CH-5′^{a}), 2.98 ppm (dd, *J* = 7.6, 11.1 Hz, 1 H, CH-5′^{b}).

^{2}H NMR (700 MHz, DMSO-d6, 30 °C): δ = 8.16 (s, 0.34 H, D-8), 7.97 ppm (s, 0.82 H, D-2).

^{13}C NMR (176 MHz, DMSO-d6, 30 °C): δ = 163.36 (C=O), 143.94 (C2), 136.99 (C4), 134.39 (C8), 134.16 (t, *J* = 38 Hz, C8-D), 120.27 (C5), 85.36 (C1′), 84.29 (C4′), 73.49 (C2′), 61.07 (C3′), 59.09 ppm (C5′).

^{15}N NMR (71 MHz, DMSO-d6, 30 °C): δ = 265.97 (N1), 252.28 (N7), 174.85 (N9), 135.43 ppm (N3).

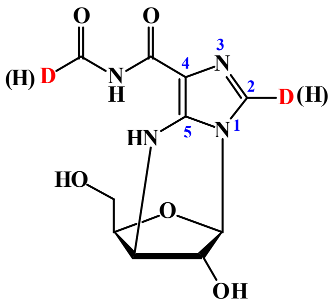

Nucleoside 14: HPLC: RT 11.48, purity 98.1%. UV/Vis (D_2O, appeared pH 4.1): λ_{max}, nm (ε, $M^{-1}cm^{-1}$): 215(17600), 310 (15000), λ_{min}, nm (ε, $M^{-1}cm^{-1}$): 264 (1800). HRMS (ESI^-): (*m/z*) $[(M + H)^+]$ - calculated for $C_{10}H_{13}N_4O_5$ 269.0880, found 269.0896; calculated for $C_{10}H_{12}DN_4O_5$ 270.0943, found 270.0972; calculated for $C_{10}H_{11}D_2N_4O_5$ 271.1006, found 271.1035.

^{1}H NMR (700 MHz, DMSO-d6, 30 °C): δ = 10.11 (s, 1 H, CO-NH), 9.11 (s, 0.1 H, C(O)H), 7.28 (s, 0.19 H, H-2), 7.11 (d, *J* = 4.7 Hz, 1H, C5-NH), 6.03 (s, 1 H, OH-2′), 5.63 (br. d, *J* = 0.25 Hz, H-1′), 4.74 (br. sign, 1 H, OH-5′), 4.32 (dt, *J* = 3.3, 6.7, 6.7 Hz, 1 H, H-4′), 3.77 (m, 1 H, H-3′), 3.56 (dd, *J* = 6.7, 11.3 Hz, 1 H, $H_{5'a}$), 3.49 ppm (dd, *J* = 6.9, 10.8 Hz, 1 H, $H_{5'b}$).

^{2}H NMR (700 MHz, DMSO-d6, 30 °C): δ = 9.11 (s, 0.9 H, C(O)H), 7.28 ppm (s, 0.91 H, D-2).

^{13}C NMR (176 MHz, DMSO-d6, 30 °C): δ = 162.29 (s, 0.1C, (C=O)H), 162.09 (t, *J* = 30.1 Hz, 0.1C, (C=O)H), 161.74 (s, 1C, C=O), 143.29 (C5), 128.87 (s, 0.19C, C2-H), 128.66 (t, *J* = 33.2 Hz, 0.81C, C2-D), 108.65 (C4), 85.87 (C4′), 84.02 (C1′), 72.57 (C2′), 60.10 (C5′), 55.97 ppm (C3′). ^{15}N NMR (71 MHz, DMSO-d6, 30 °C): δ = 250.33 (N3), 179.25 (N1), 158.81 (CO-NH), 64.28 ppm (C5-NH).

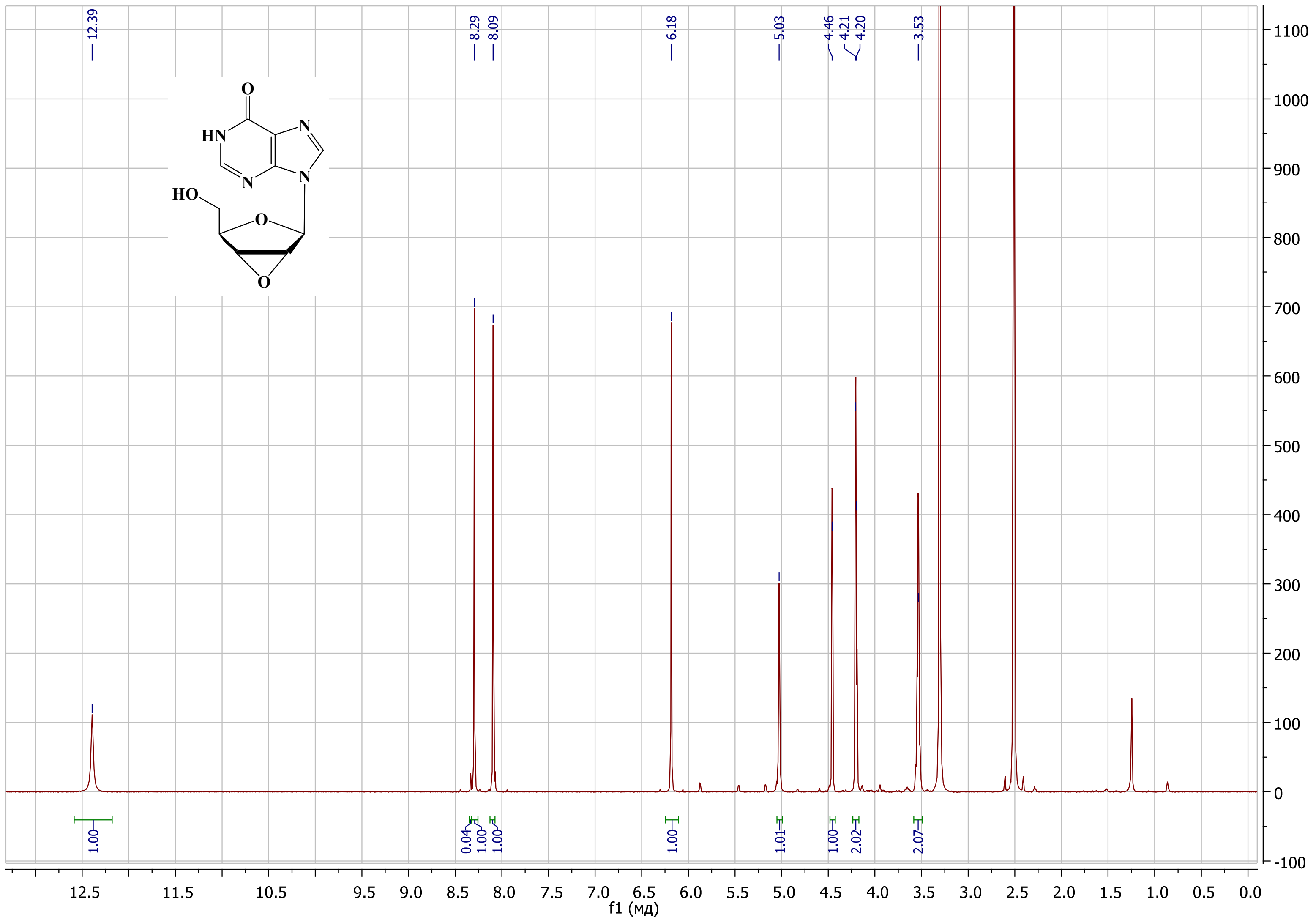

The ^{1}H NMR spectrum of 2',3'-anhydroinosine **11** (DMSO-d6, 30 °C).

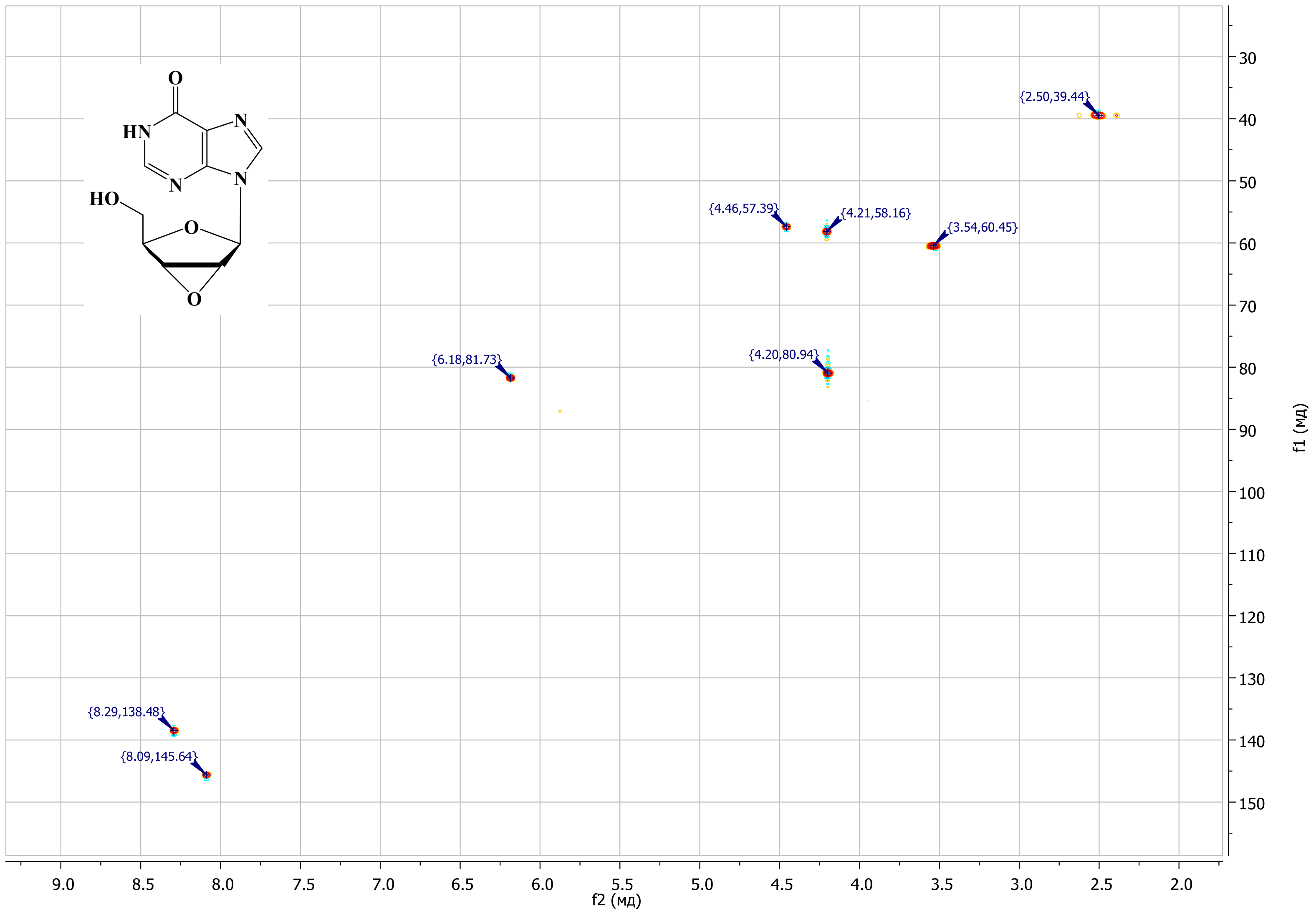

The ^{1}H-^{13}C-HSQC NMR spectrum of 2',3'-anhydroinosine **11** (DMSO-d6, 30 °C).

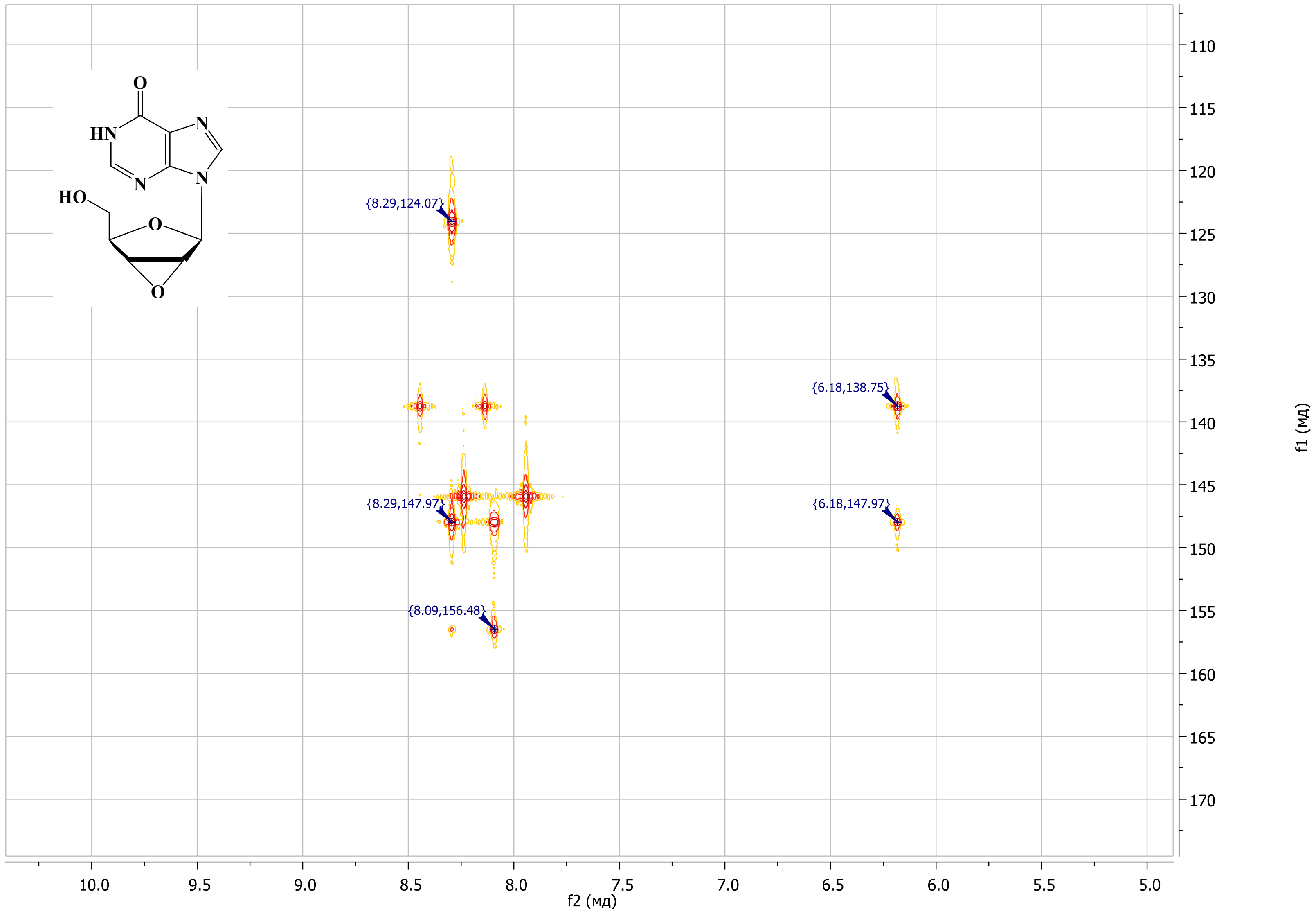

The fragment of ^{1}H-^{13}C-HMBC NMR spectrum of 2',3'-anhydroinosine **11** (DMSO-d6, 30 °C).

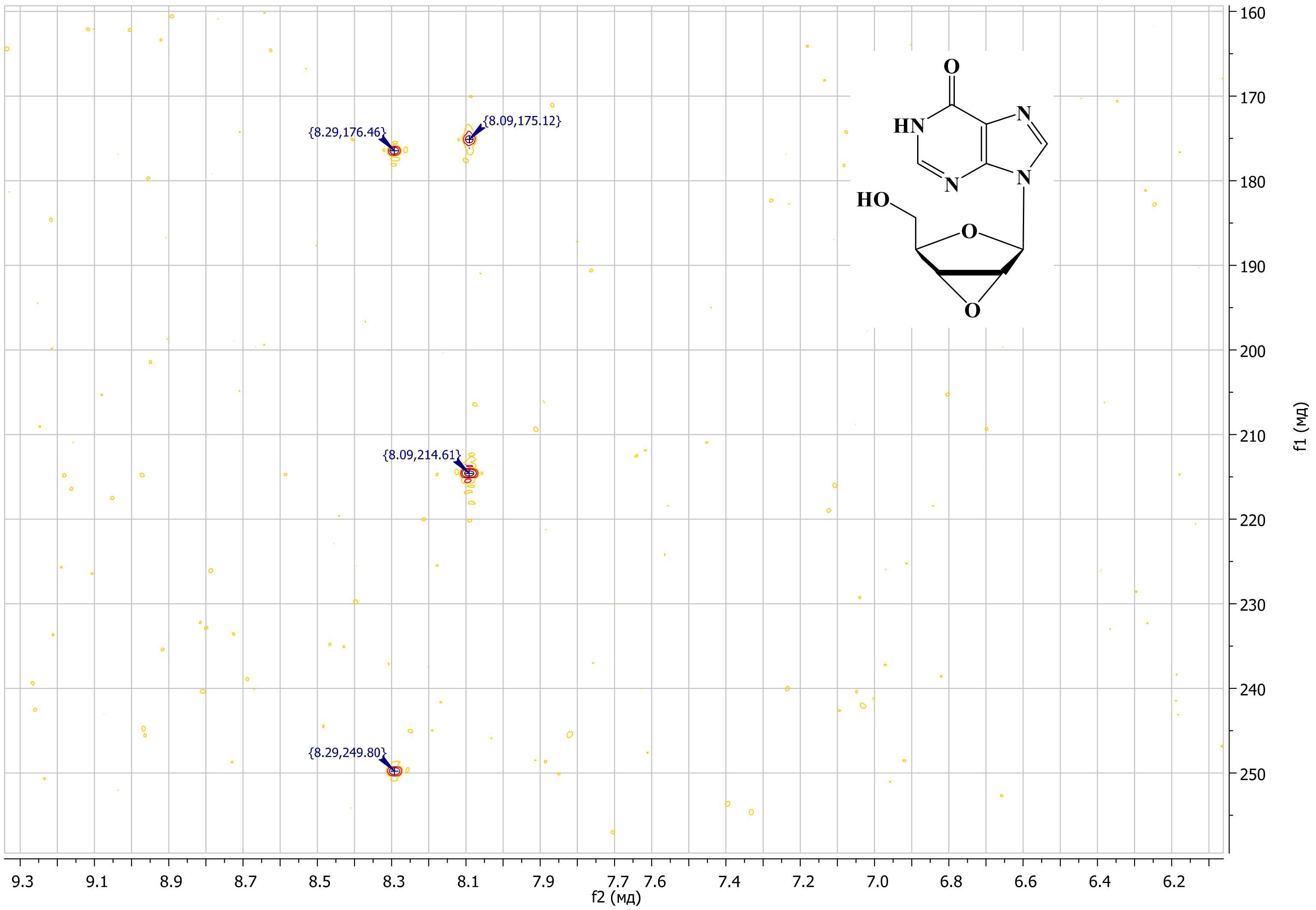

The fragment of ¹H-¹⁵N-HMBC NMR spectrum of 2',3'-anhydroinosine **11** (DMSO-d6, 30 °C)

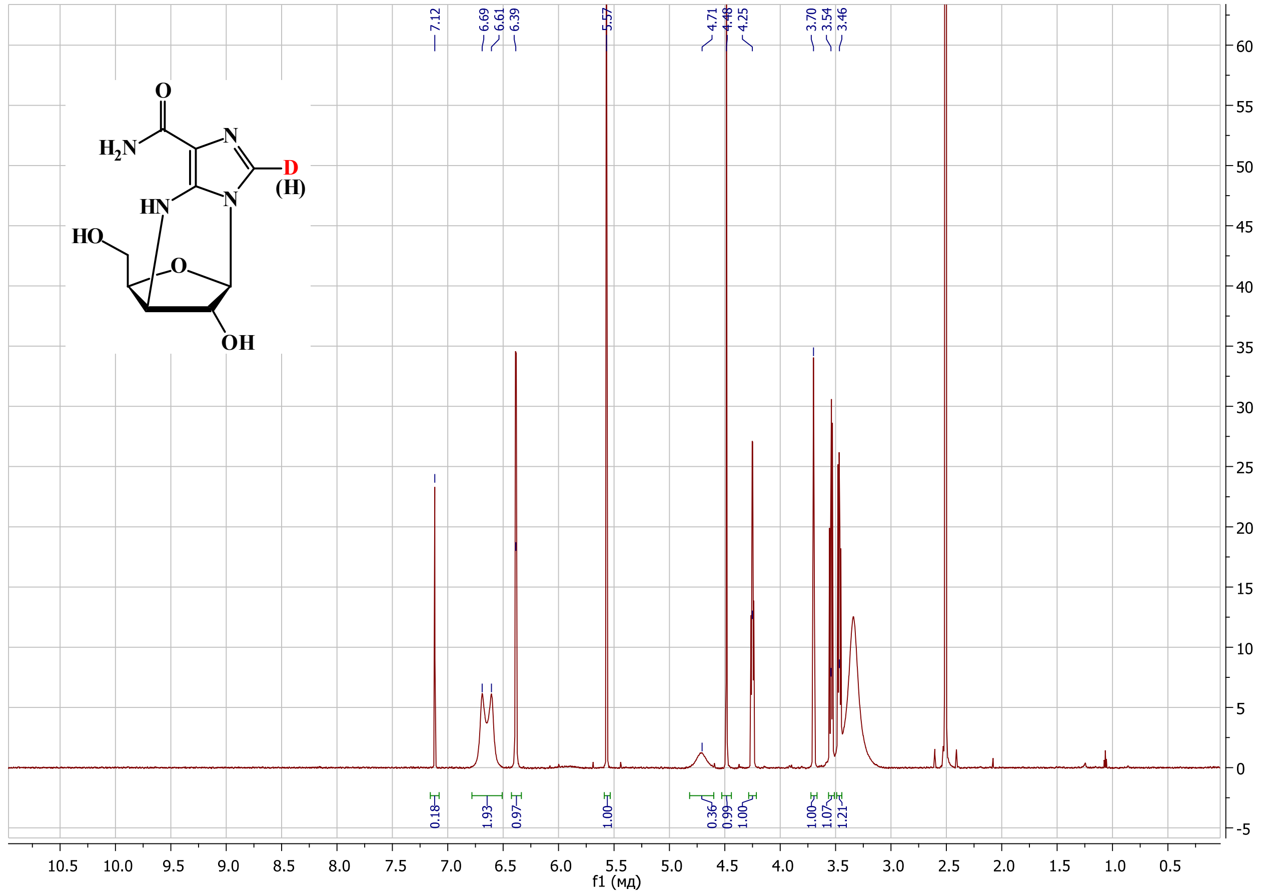

The ^{1}H NMR spectrum of compound **10** (DMSO-d6, 30 °C).

^{1}H NMR (700 MHz, DMSO-d6, 30 °C): sδ = 7.12 (s, 0.18 H, H-8), 6.69 and 6.61 (2 s, 2 H, $CONH_2$), 6.39 (d, J = 4.4 Hz, 1 H, NH), 5.57 (d, J < 0.5 Hz, 1H, H-1′), 4.71 (br. sign, 0.36 H, OH-5′), 4.48 (br. sign, 1H, H-2′), 4.25 (dt, J = 3.2, 6.6, 6.6 Hz, 1 H, H-4′), 3.70 (m, 1 H, H-3′), 3.54 (dd, J = 7.0, 11.3 Hz, 1 H, CH-5′a), 3.46 ppm (dd, J = 6.5, 11.3 Hz, 1 H, CH-5′b).

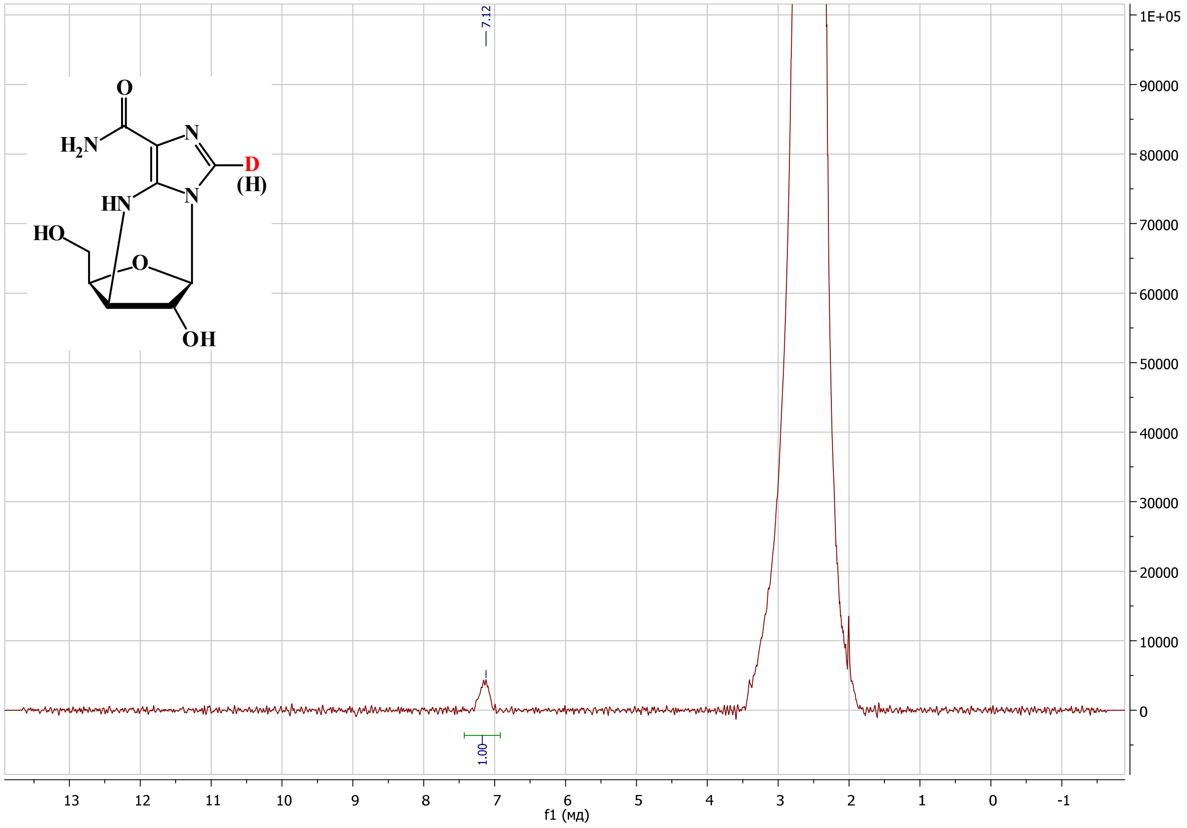

The ^{2}H NMR spectrum of compound **10** (DMSO-d6, 30 °C)

^{2}H NMR (700 MHz, DMSO-d6, 30 °C): δ = 7.12 ppm (s, D-8).

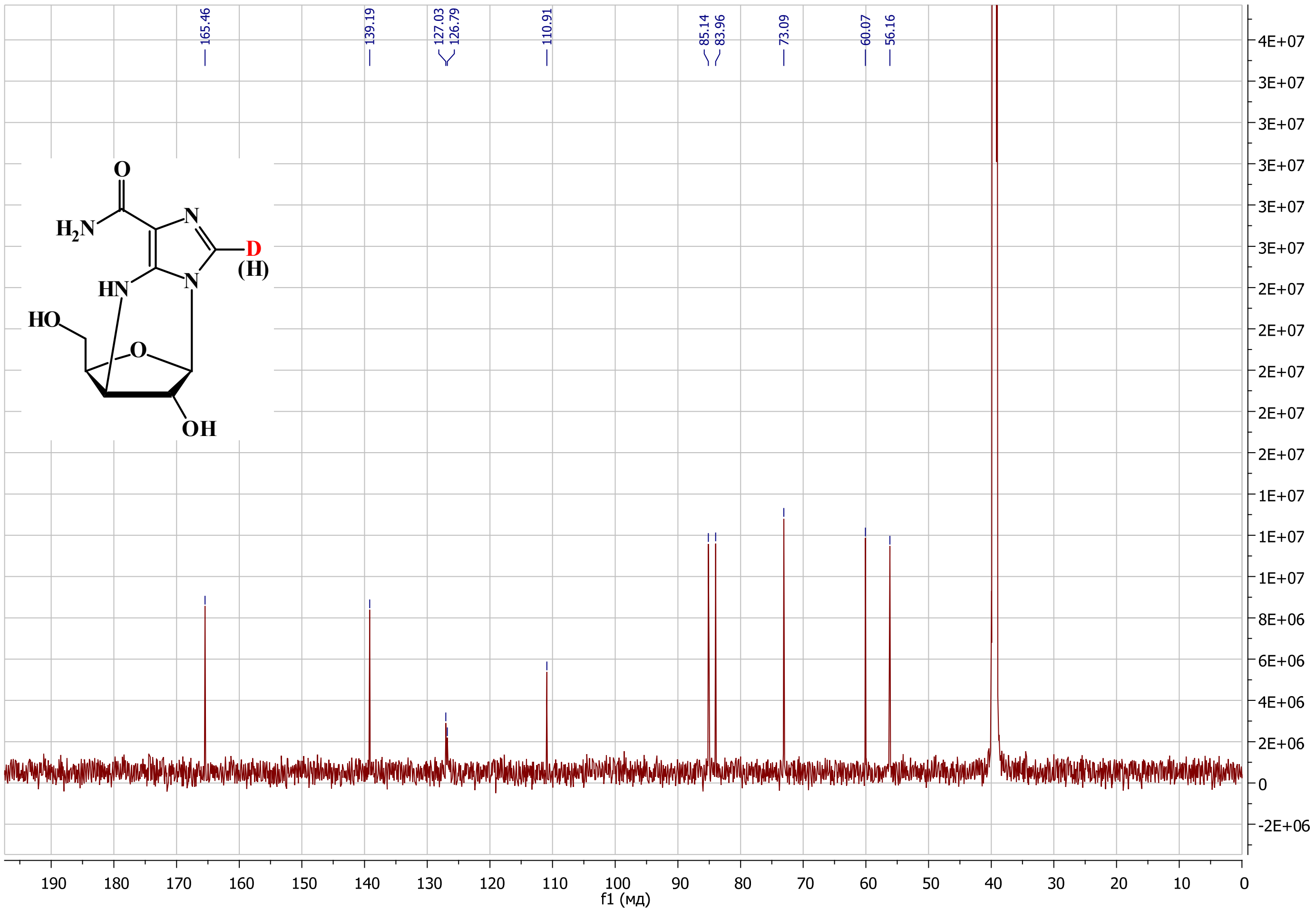

The ^{13}C NMR spectrum of compound **10** (DMSO-d6, 30 °C).

^{13}C NMR (176 MHz, DMSO-d6, 30 °C): δ = 165.46 (C=O), 139.19 (C5), 127.03(C2), 126.79 (t, J = 18.5Hz, C2-D), 110.91 (C4), 85.14 (C4′), 83.96 (C1′), 73.09 (C2′), 60.07 (C5′), 56.16 ppm (C3′).

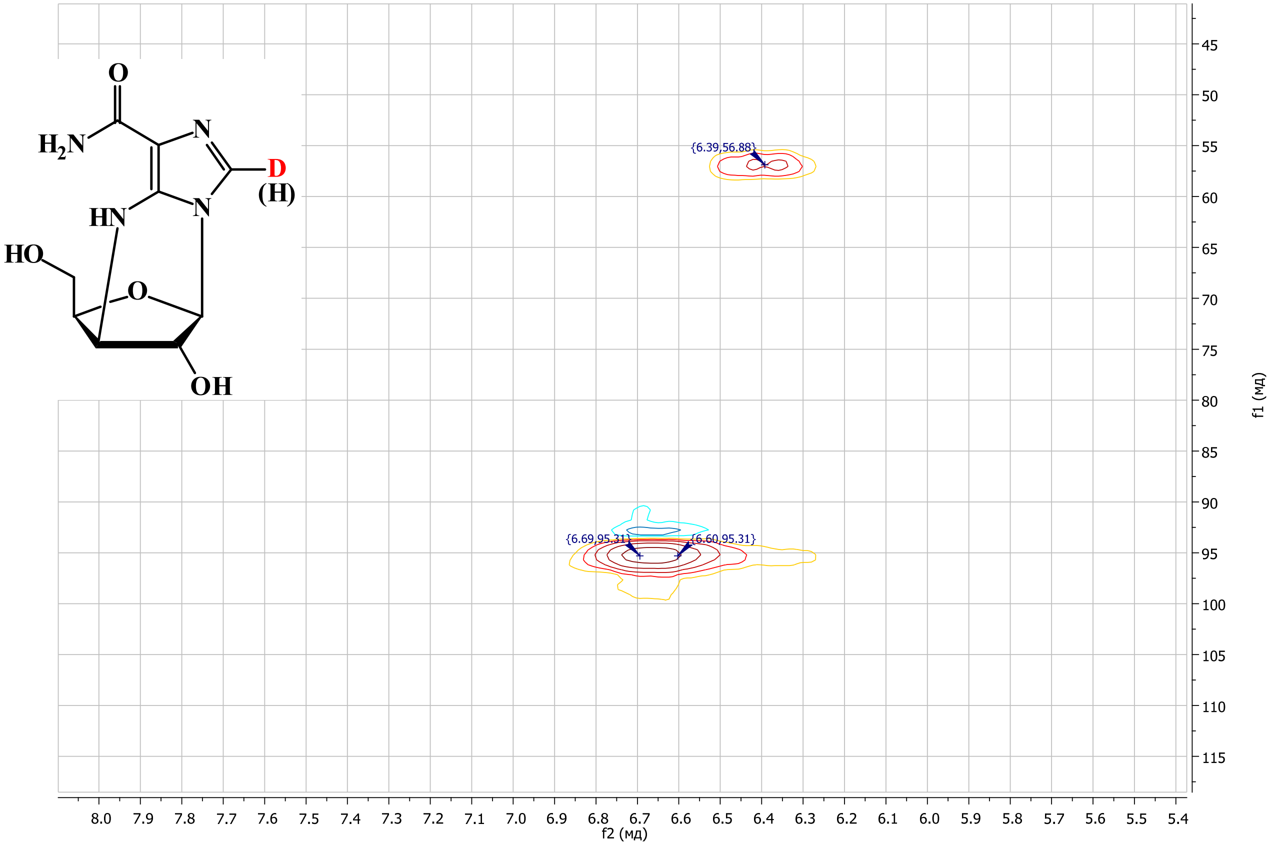

The fragment of ^{1}H-^{15}N-HSQC NMR spectrum of nucleoside **10** (DMSO-d6, 30 °C).

^{15}N NMR (71 MHz, DMSO-d6, 30 °C): δ = 253.35 (N3), 177.94 (N1), 95.31 (NH_2), 56.88 ppm (NH).

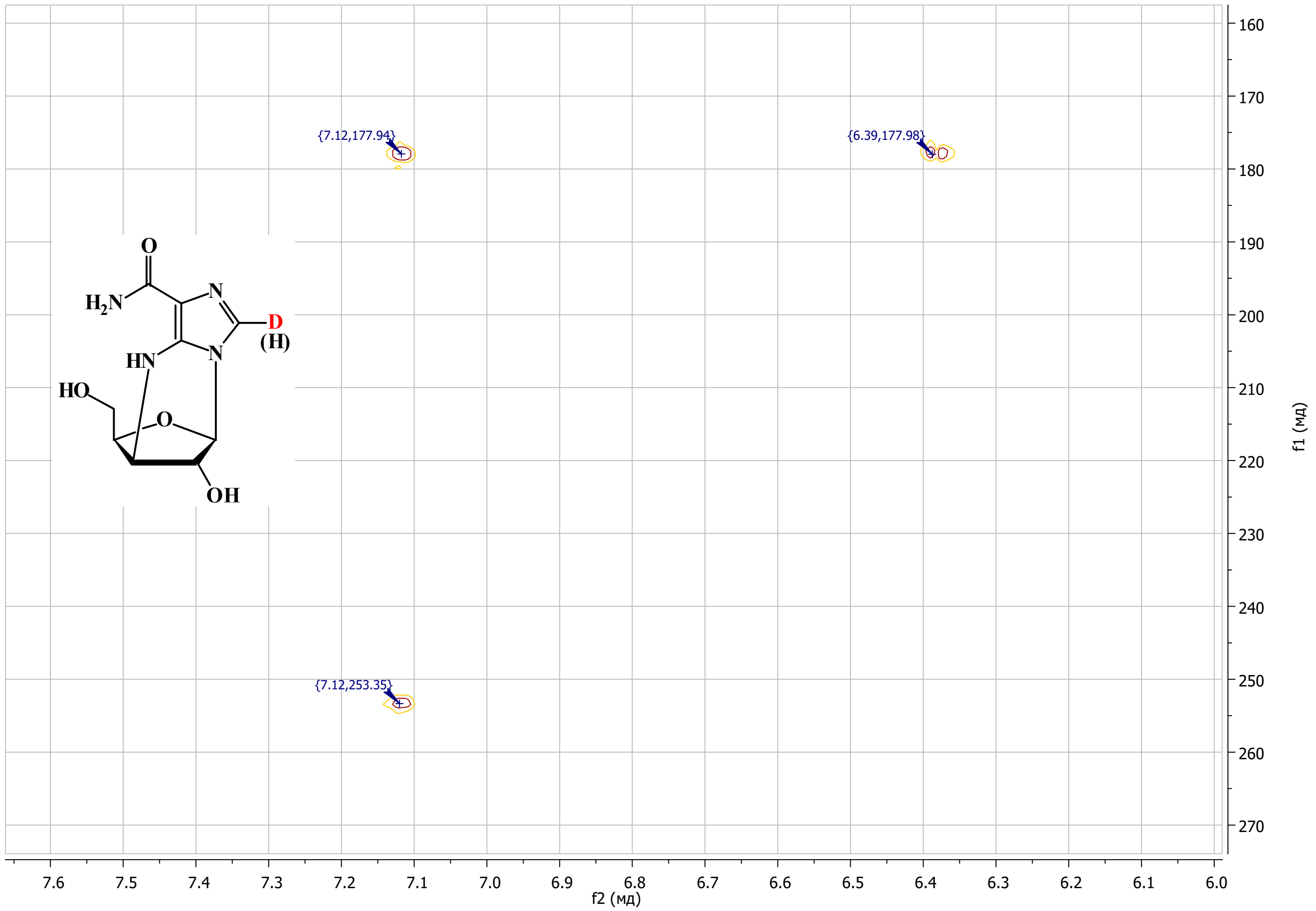

The fragment of ^{1}H-^{15}N-HMBC NMR spectrum of nucleoside **10** (DMSO-d6, 30 °C).

^{15}N NMR (71 MHz, DMSO-d6, 30 °C): δ = 253.35 (N3), 177.94 (N1), 95.31 (NH_2), 56.88 ppm (NH).

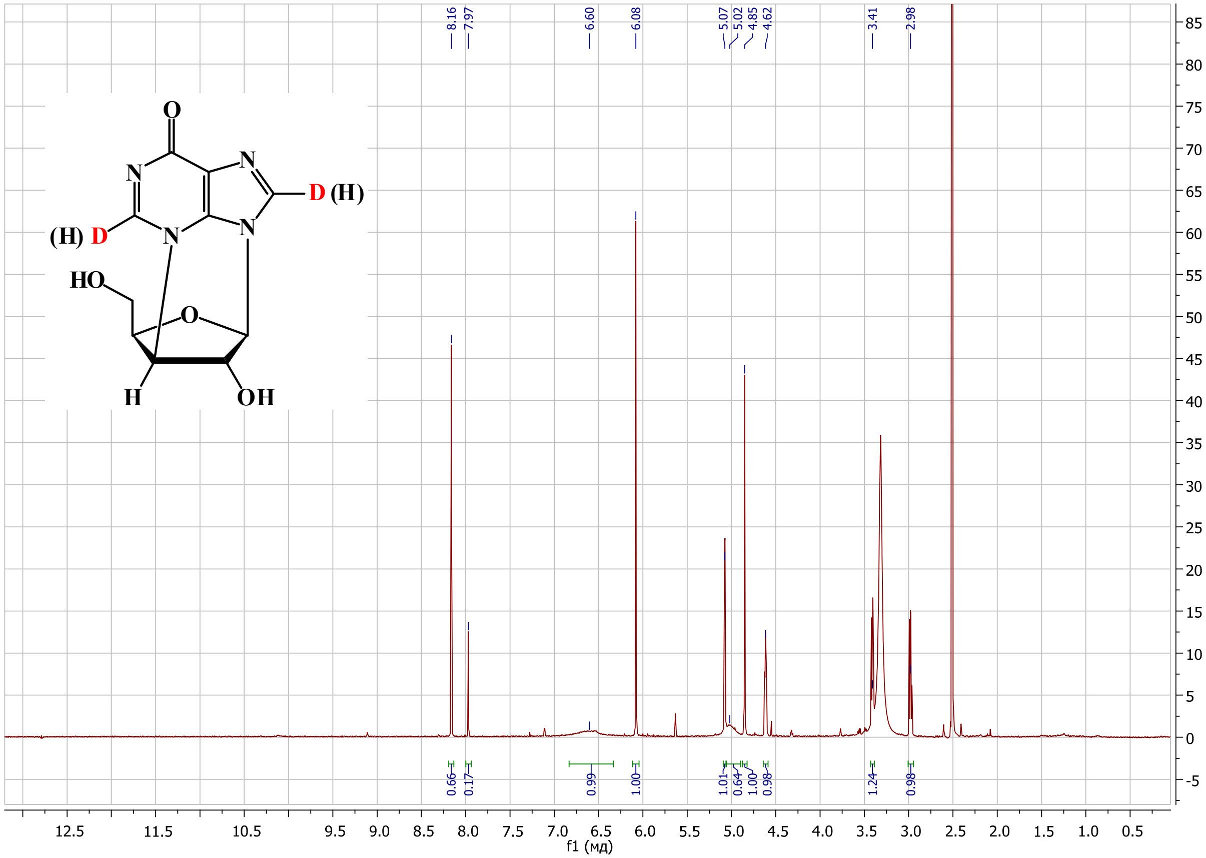

The ^{1}H NMR spectrum of compound **12** (DMSO-d6, 30 °C).

^{1}H NMR (700 MHz, DMSO-d6, 30 °C): δ = 8.16 (s, 0.66 H, H-8), 7.97 (s, 0.18 H, H-2), 6.08 (1H, d, J < 1 Hz, H-1′), 5.07 (d, J = 3.1 Hz, 1 H, H-3′), 4.85 (m,1 H, J < 1 Hz, H-2′), 4.62 (ddd, J = 3.4, 6.1, 7.5 Hz.0, 1 H, H-4′), 3.41 (dd, J = 6.1, 11.1 Hz, 1 H, CH-5′[a]), 2.98 ppm (dd, J = 7.6, 11.1 Hz, 1 H, CH-5′[b]).

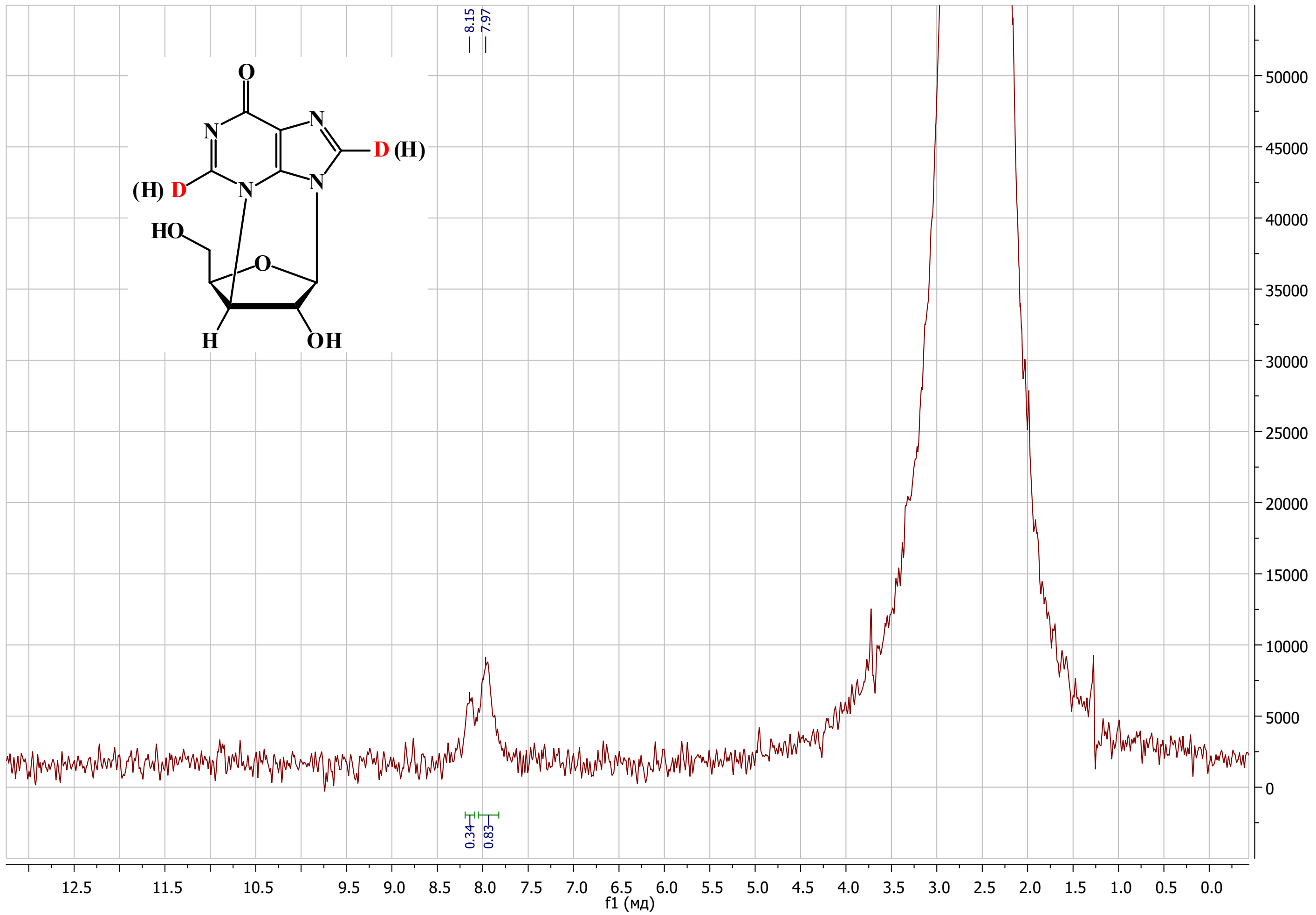

The ^{2}H NMR spectrum of compound **12** (DMSO-d6, 30 °C).

^{2}H NMR (700 MHz, DMSO-d6, 30 °C): δ = 8.16 (s, 0.34 H, D-8), 7.97 ppm (s, 0.82 H, D-2).

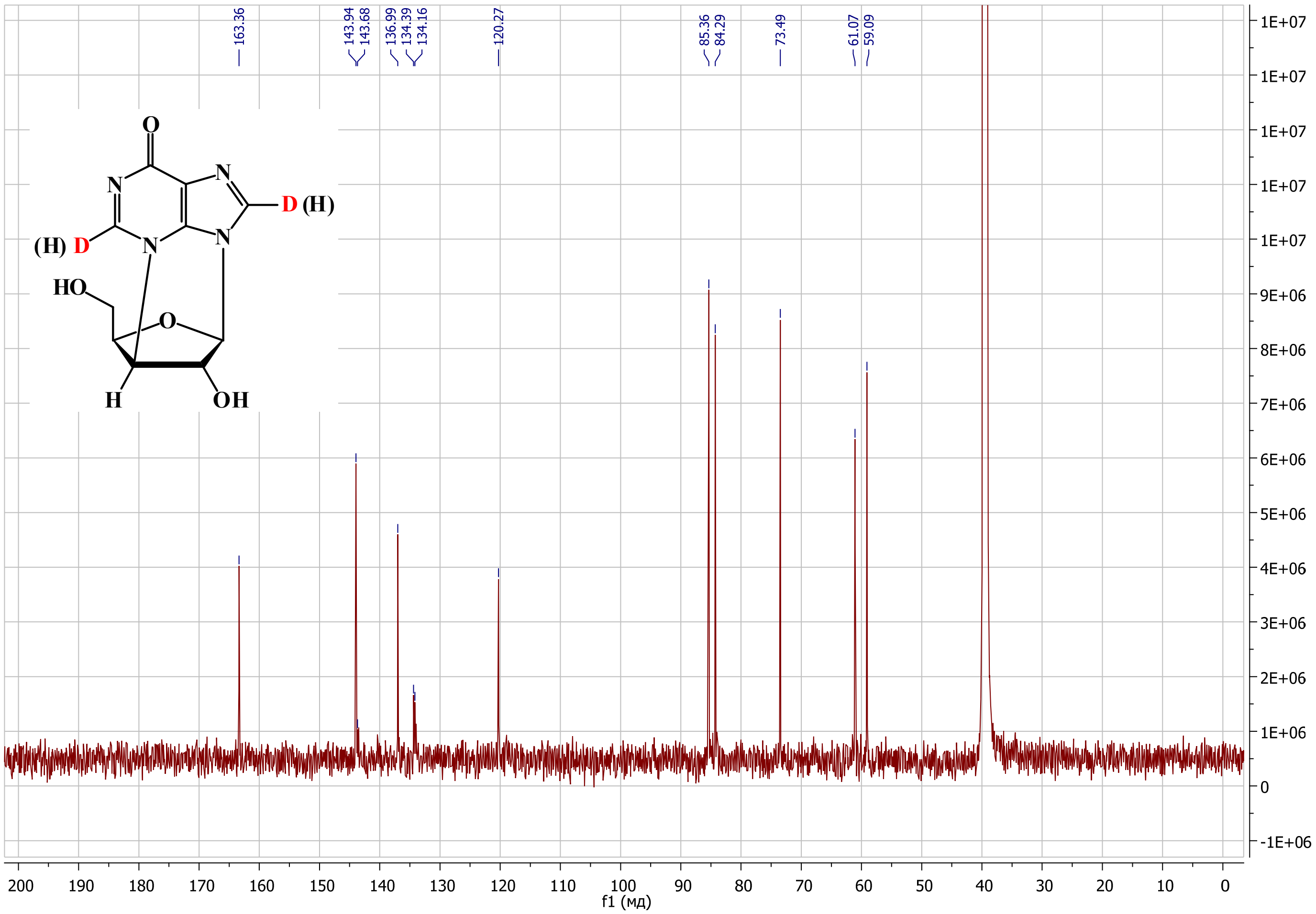

The ^{13}C NMR spectrum of compound **12**)(DMSO-d6, 30 °C).

^{13}C NMR (176 MHz, DMSO-d6, 30 °C): δ = 163.36 (C=O), 143.94 (C2), 136.99 (C4), 134.39 (C8), 134.16 (t, *J* = 38 Hz, C8-D), 120.27 (C5), 85.36 (C1’), 84.29 (C4’), 73.49 (C2’), 61.07 (C3’), 59.09 ppm (C5’).

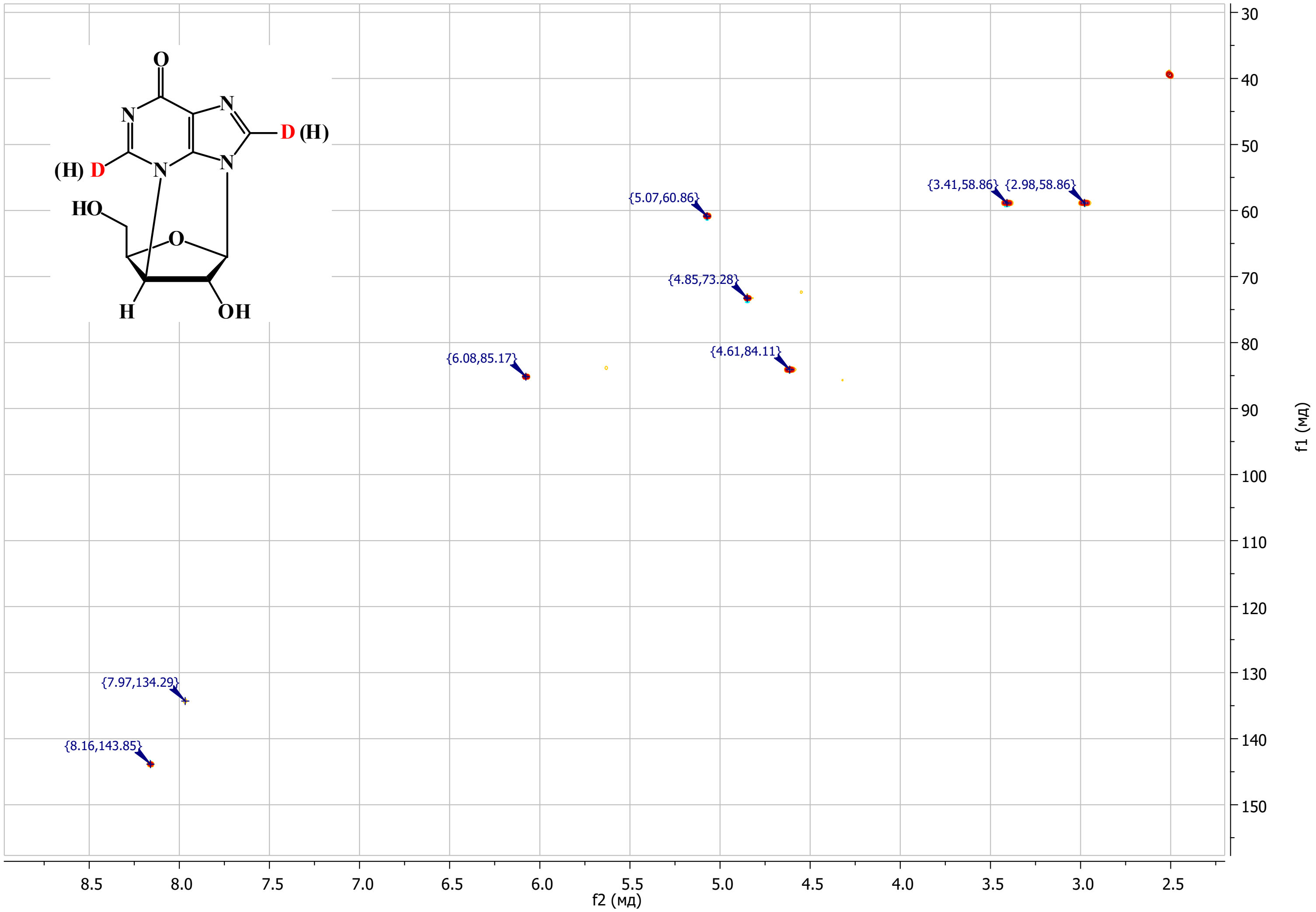

The fragment of ^{1}H-^{13}C-HSQC NMR spectrum of nucleoside **12** (DMSO-d6, 30 °C).

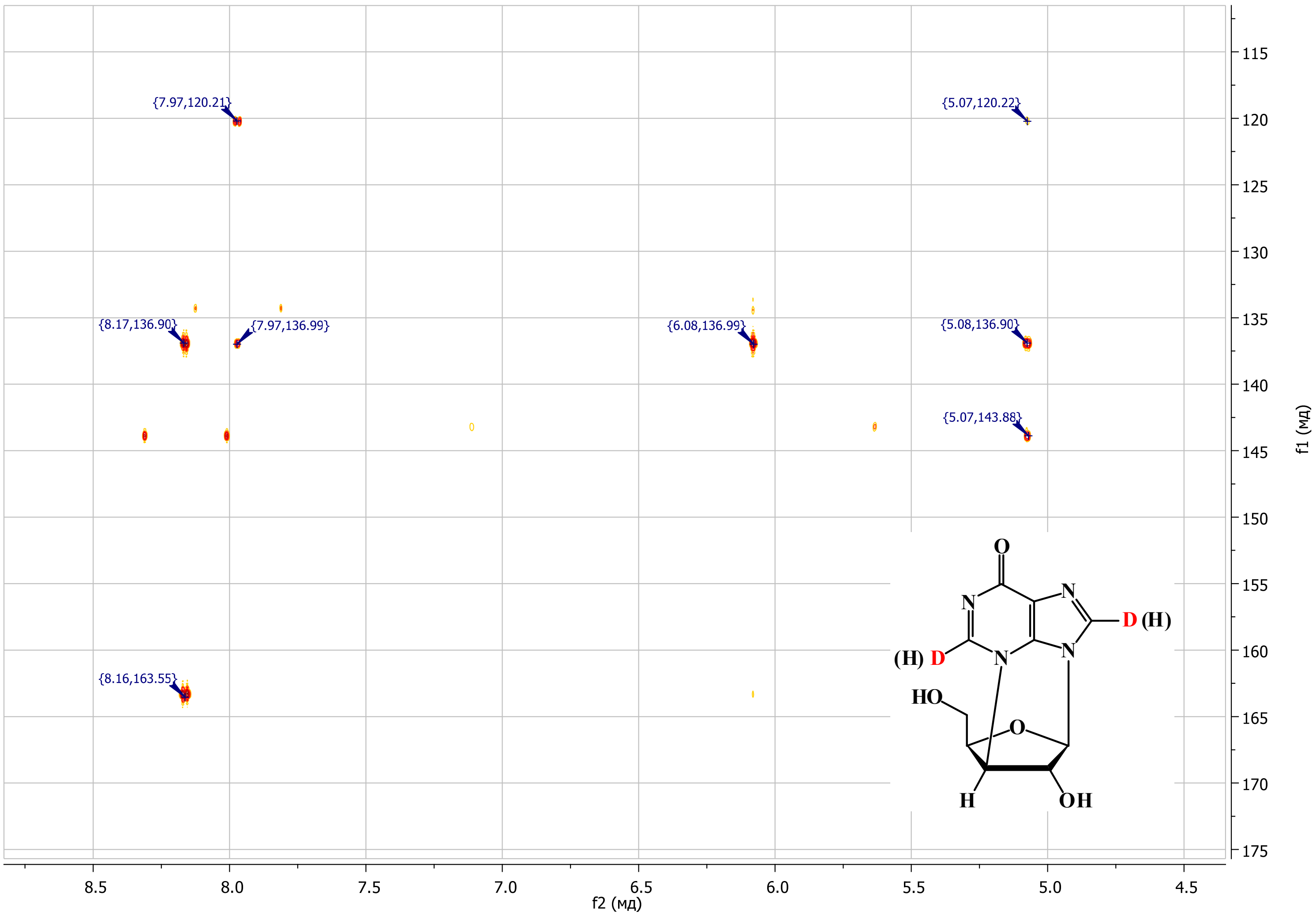

The fragment of ^{1}H-^{13}C-HMBC NMR spectrum of nucleoside **12** (DMSO-d6, 30 °C).

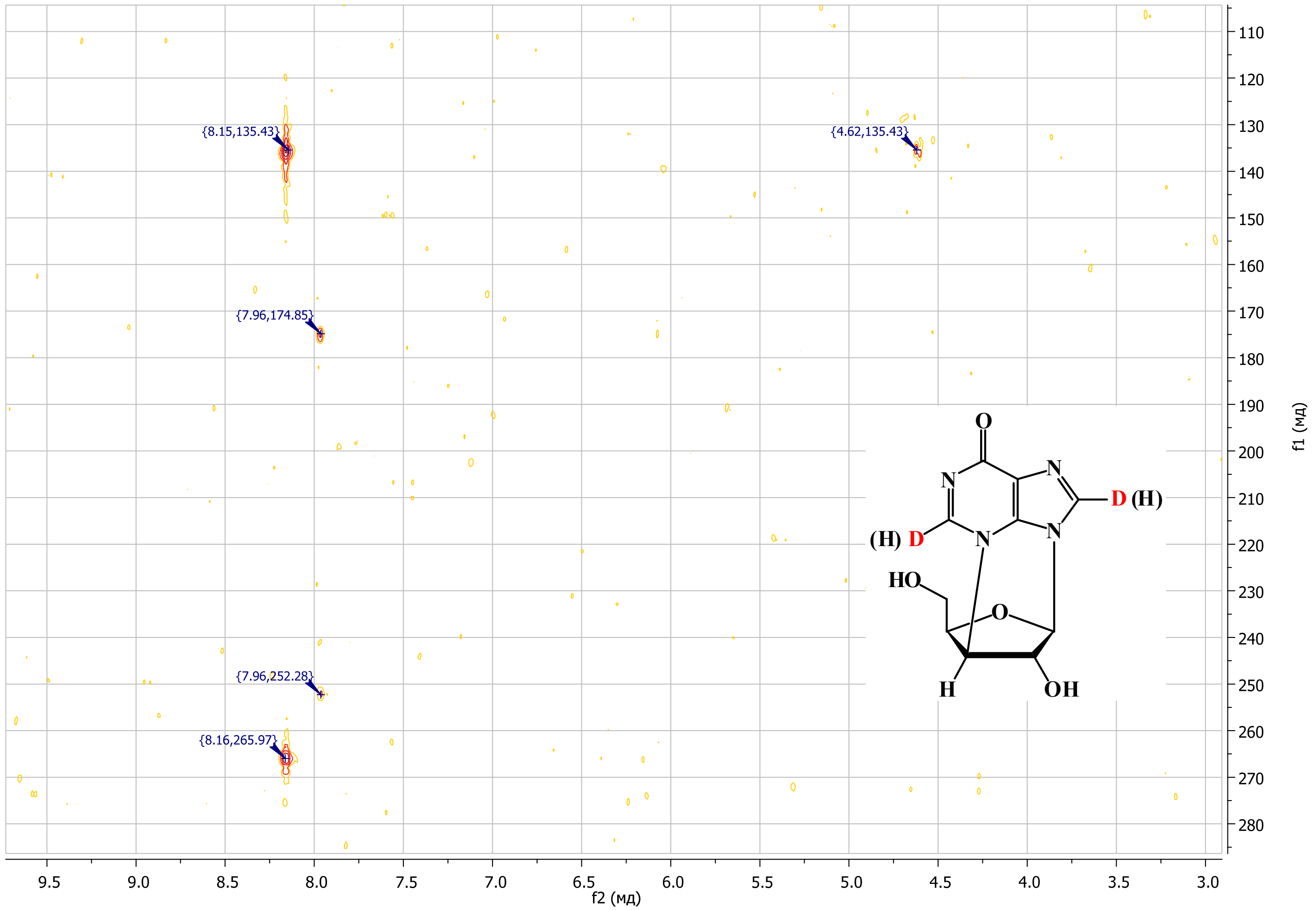

The fragment of ^{1}H-^{15}N-HMBC NMR spectrum of nucleoside **12** (DMSO-d6, 30 °C).

^{15}N NMR (71 MHz, DMSO-d6, 30 °C): δ = 265.97 (N1), 252.28 (N7), 174.85 (N9), 135.43 ppm (N3).

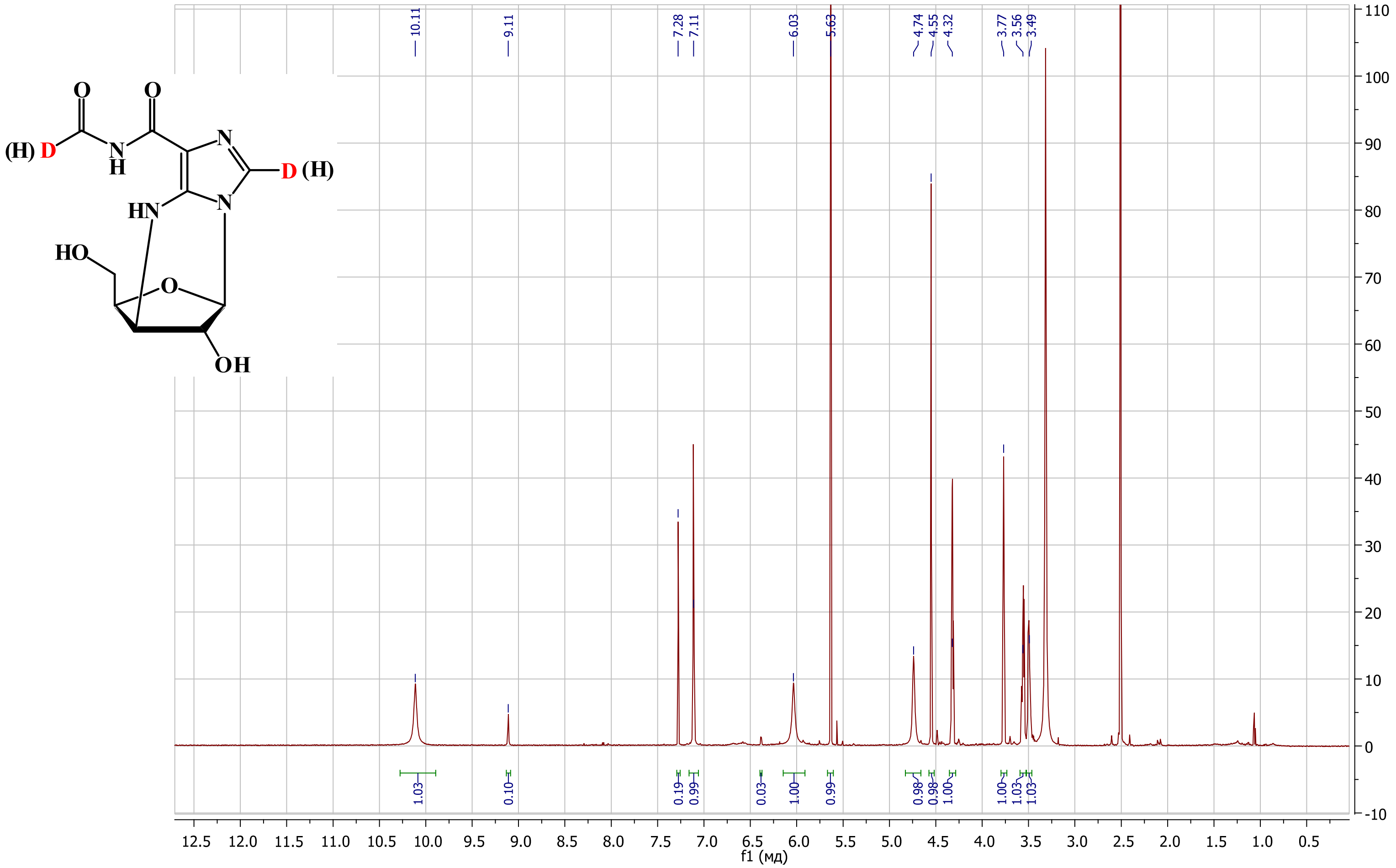

The ^{1}H NMR spectrum of compound **14** (DMSO-d6, 30 °C).

^{1}H NMR (700 MHz, DMSO-d6, 30 °C): δ = 10.11 (s, 1 H, CO-NH), 9.11 (s, 0.1 H, C(O)H), 7.28 (s, 0.19 H, H-2), 7.11 (d, *J* = 4.7 Hz, 1H, C5-NH), 6.03 (s, 1 H, OH-2′), 5.63 (br. d, *J* = 0.25 Hz, H-1′), 4.74 (br. sign, 1 H, OH-5′), 4.32 (dt, *J* = 3.3, 6.7, 6.7 Hz, 1 H, H-4′), 3.77 (m, 1 H, H-3′), 3.56 (dd, *J* = 6.7, 11.3 Hz, 1 H, $H_{5'a}$), 3.49 ppm (dd, *J* = 6.9, 10.8 Hz, 1 H, $H_{5'b}$).

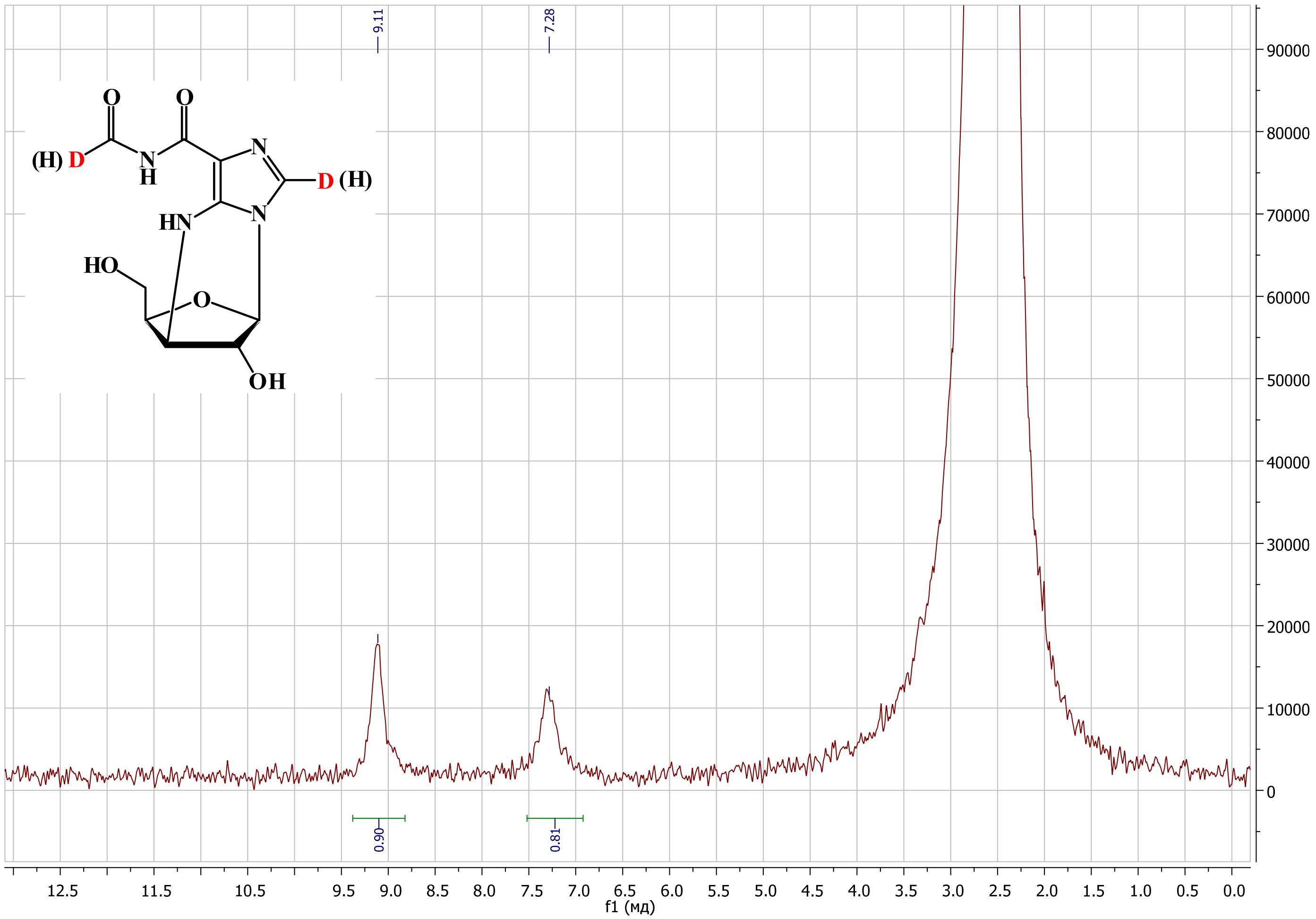

The ^{2}H NMR spectrum of compound **14** (DMSO-d6, 30 °C).

^{2}H NMR (700 MHz, DMSO-d6, 30 °C): δ = 9.11 (s, 0.9 H, C(O)H), 7.28 ppm (s, 0.91 H, D-2).

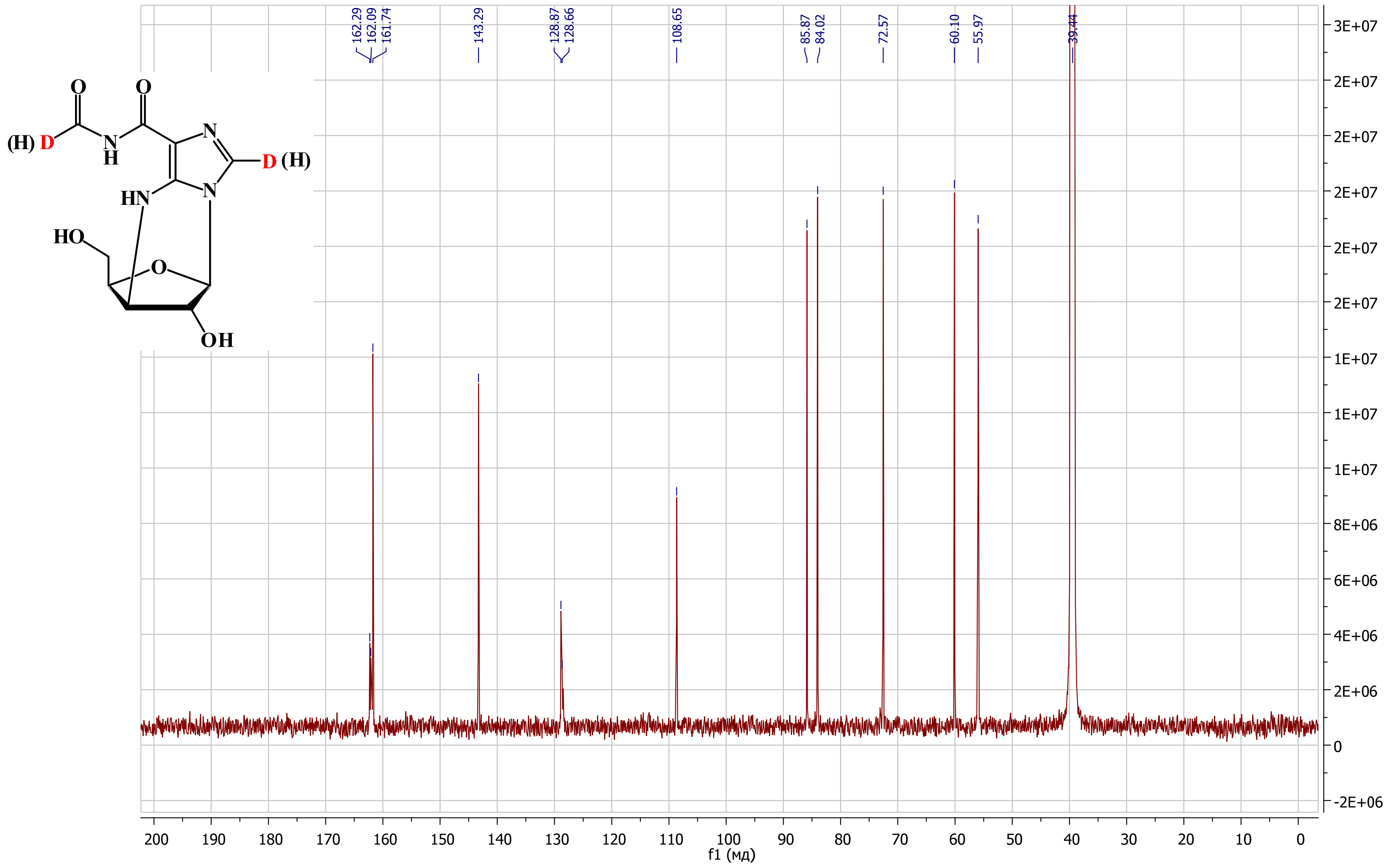

The ^{13}C NMR spectrum of compound **14** (DMSO-d6, 30 °C).

^{13}C NMR (176 MHz, DMSO-d6, 30 °C): δ = 162.29 (s, 0.1C, (C=O)H), 162.09 (t, *J* = 30.1 Hz, 0.1C, (C=O)H), 161.74 (s, 1C, C=O), 143.29 (C5), 128.87 (s, 0.19C, C2-H), 128.66 (t, *J* = 33.2 Hz, 0.81C, C2-D), 108.65 (C4), 85.87 (C4′), 84.02 (C1′), 72.57 (C2′), 60.10 (C5′), 55.97 ppm (C3′).

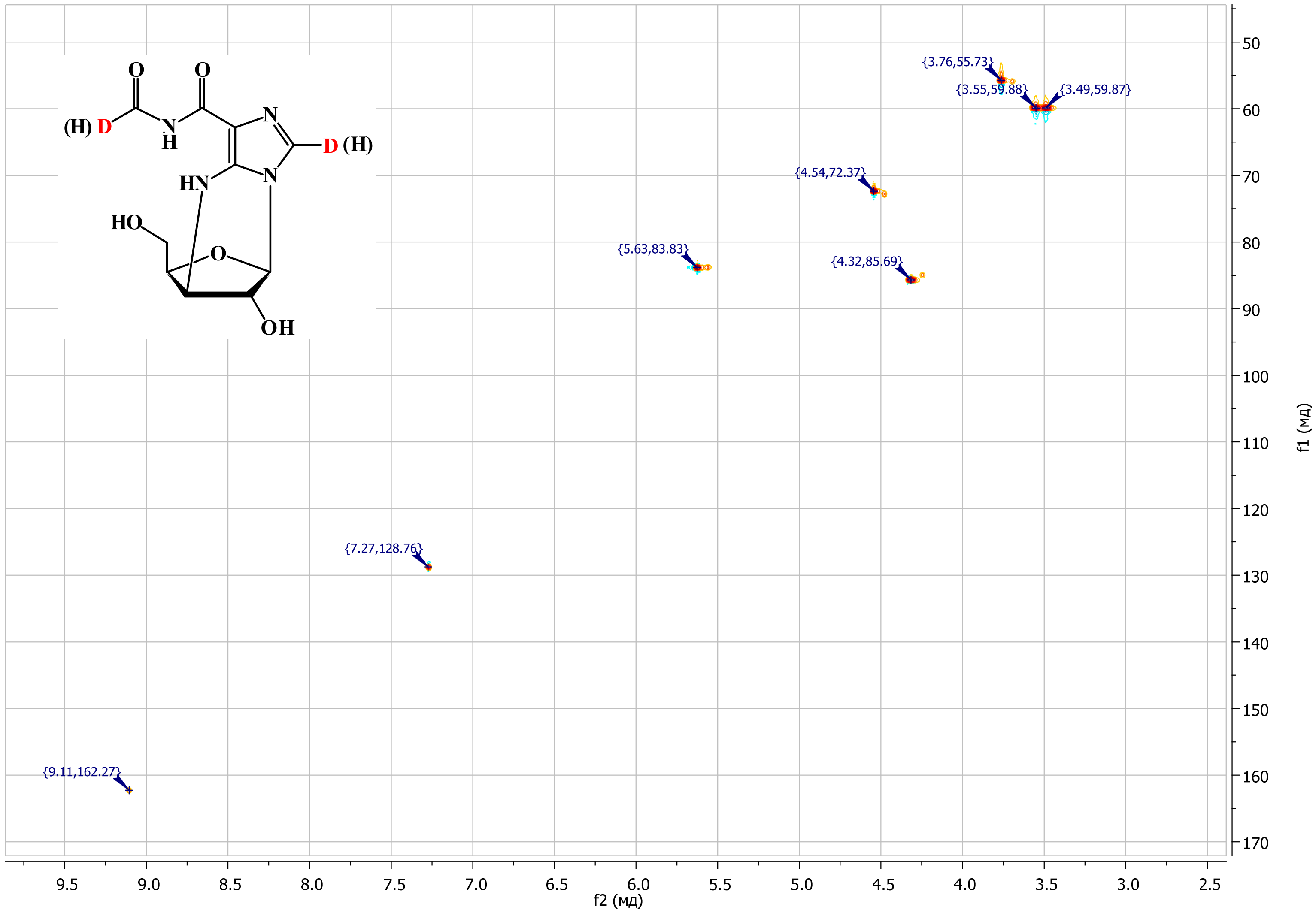

The fragment of ^{1}H-^{13}C-HSQC NMR spectrum of nucleoside **14** (DMSO-d6, 30 °C).

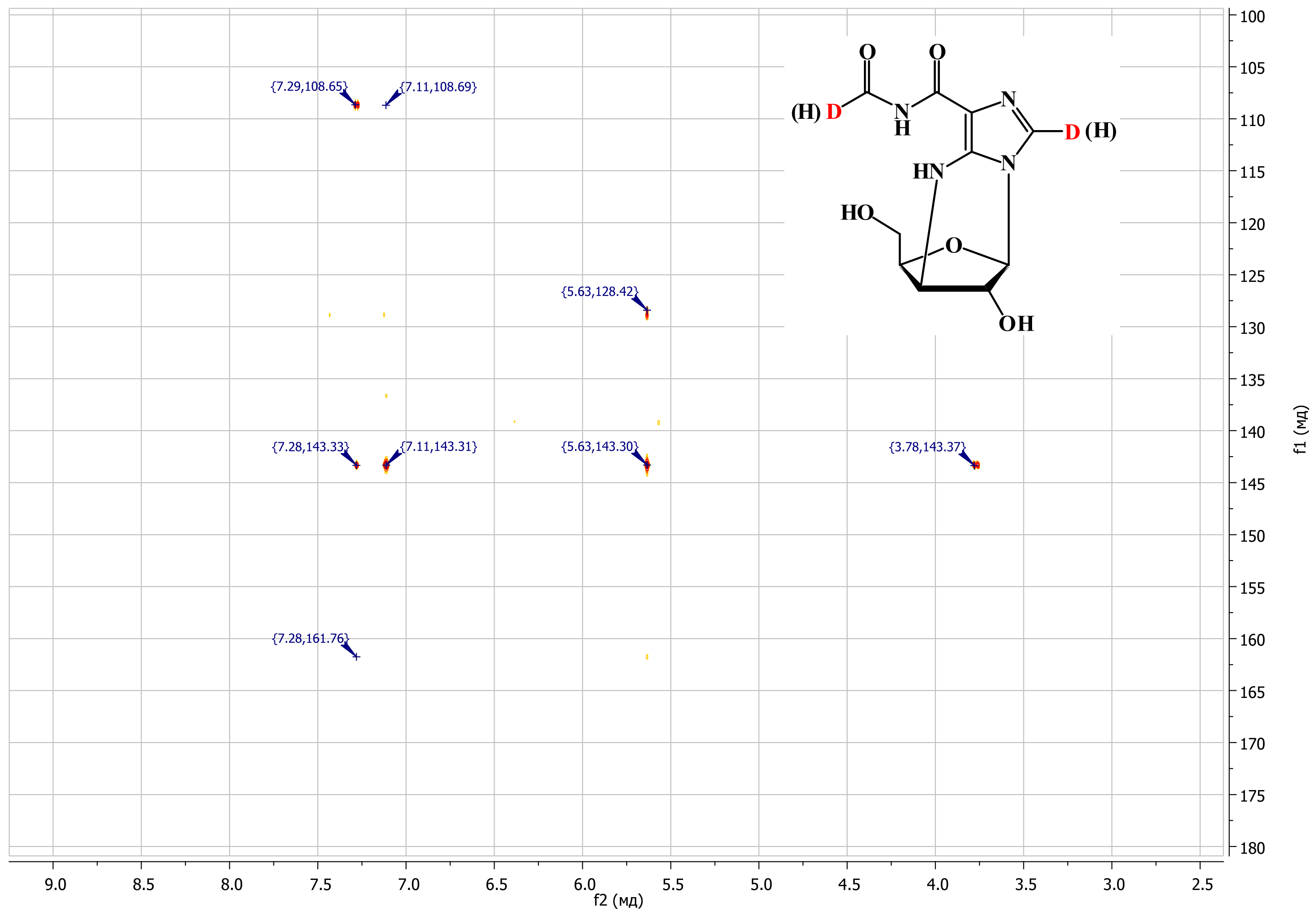

The fragment of ^{1}H-^{13}C-HMBC NMR spectrum of nucleoside **14** (DMSO-d6, 30 °C).

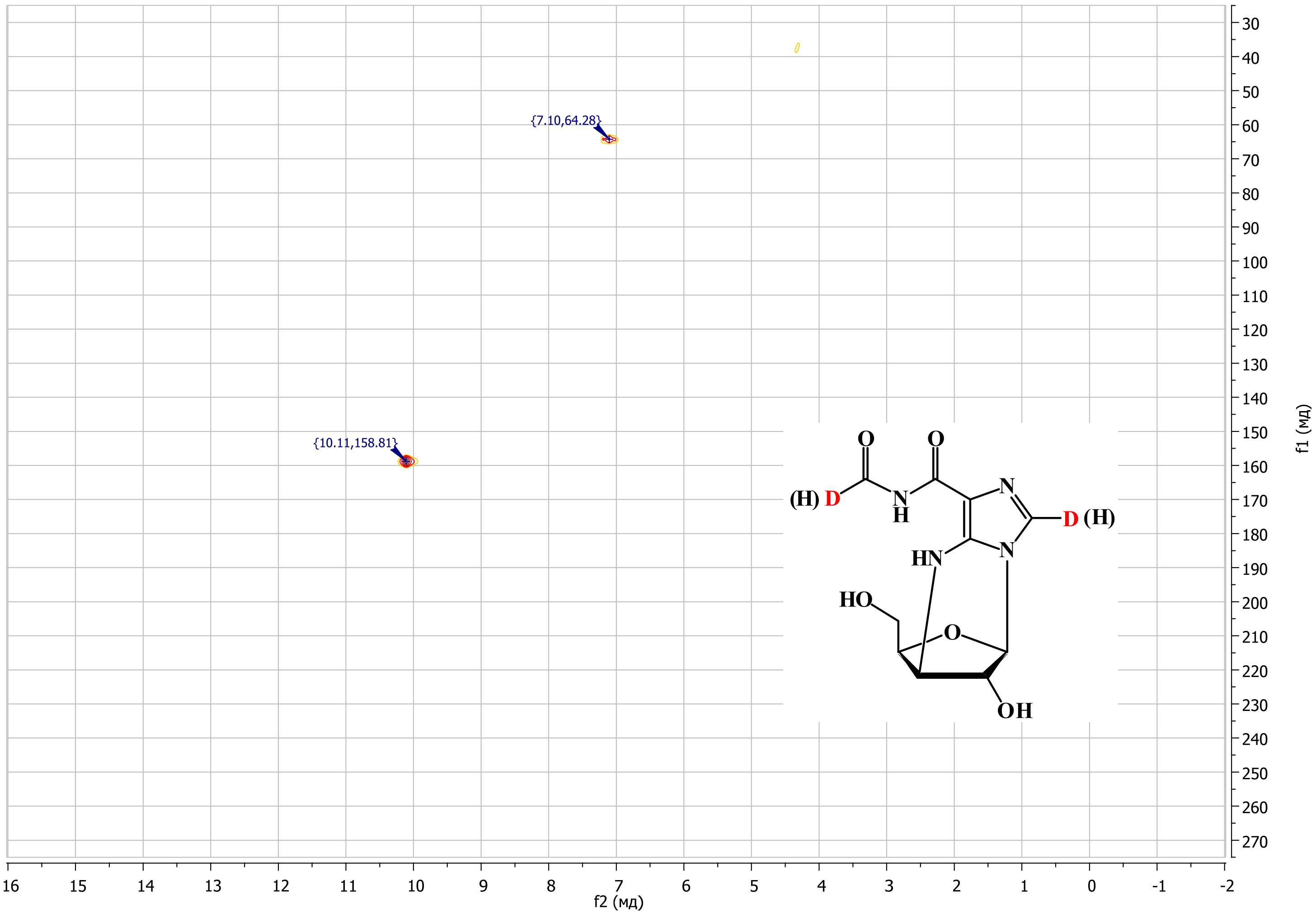

The fragment of ^{1}H-^{15}N-HSQC NMR spectrum of nucleoside **14** (DMSO-d6, 30 °C).

^{15}N NMR (71 MHz, DMSO-d6, 30 °C): δ = 250.33 (N3), 179.25 (N1), 158.81 (CO-NH), 64.28 ppm (C5-NH).

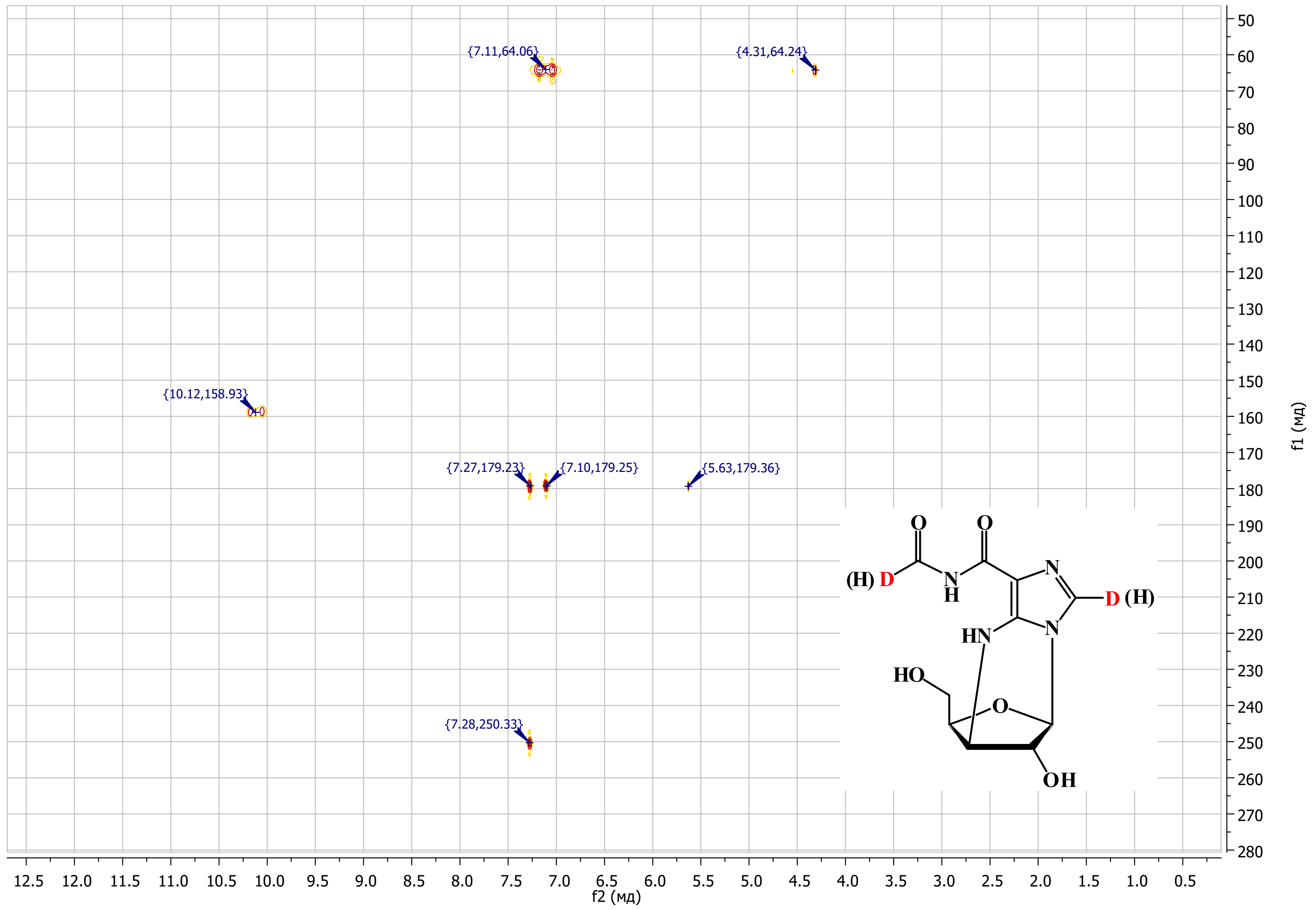

The fragment of ^{1}H-^{15}N-HMBC NMR spectrum of nucleoside **14** (DMSO-d6, 30 °C).

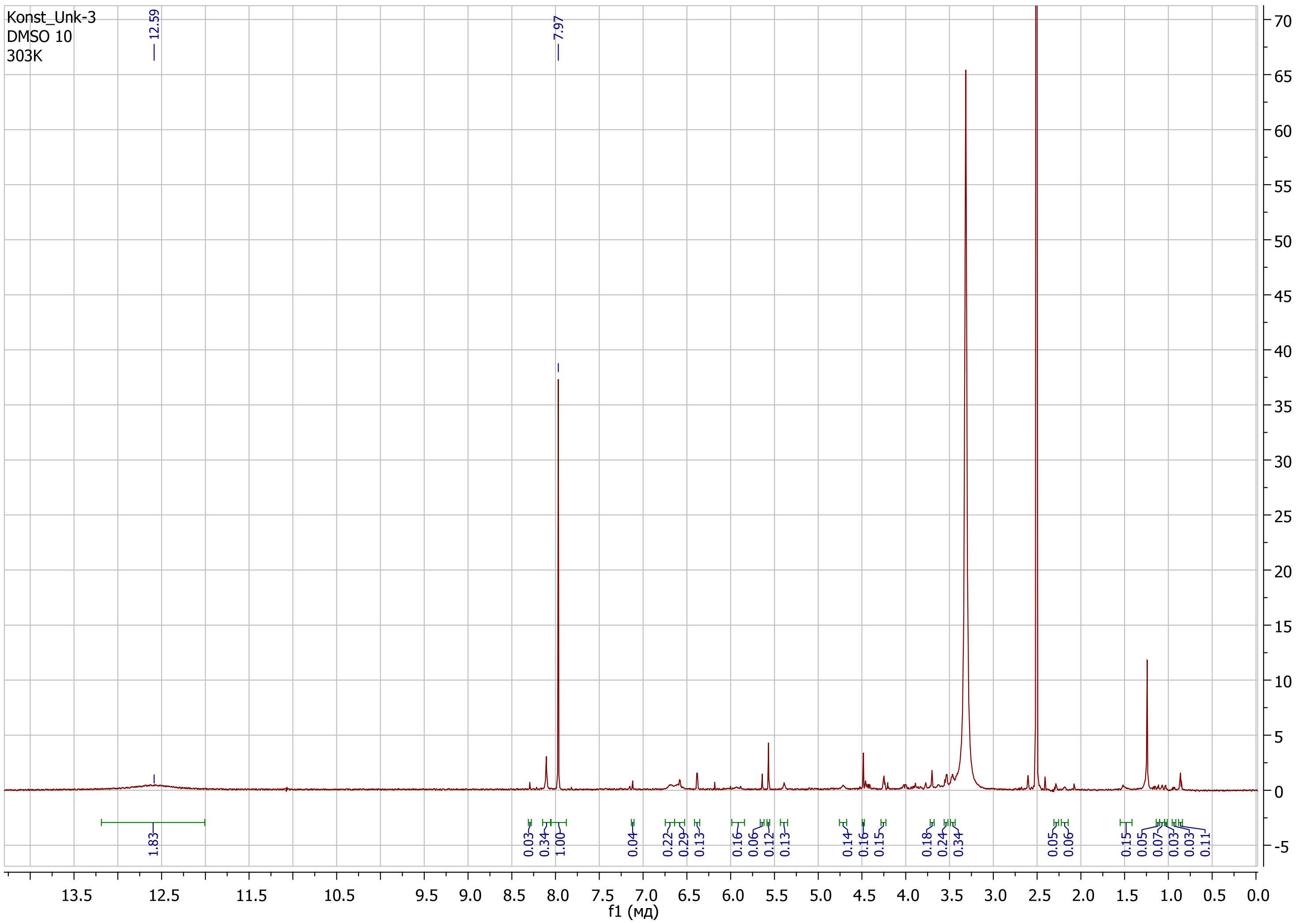

The ^{1}H NMR spectrum of compound **13** (DMSO-d6, 30 °C).
